# Novel Findings from CNVs Implicate Inhibitory and Excitatory Signaling Complexes in Schizophrenia

**DOI:** 10.1016/j.neuron.2015.04.022

**Published:** 2015-06-03

**Authors:** Andrew J. Pocklington, Elliott Rees, James T.R. Walters, Jun Han, David H. Kavanagh, Kimberly D. Chambert, Peter Holmans, Jennifer L. Moran, Steven A. McCarroll, George Kirov, Michael C. O’Donovan, Michael J. Owen

**Affiliations:** 1 MRC Centre for Neuropsychiatric Genetics and Genomics, Institute of Psychological Medicine and Clinical Neurosciences, Cardiff University, Cardiff CF14 4XN, UK; 2 Stanley Center for Psychiatric Research, The Broad Institute of MIT and Harvard, 75 Ames Street, Cambridge, MA 02142, USA; 3 Department of Genetics, Harvard Medical School, 77 Avenue Louis Pasteur, Boston, MA 02115, USA

## Abstract

We sought to obtain novel insights into schizophrenia pathogenesis by exploiting the association between the disorder and chromosomal copy number (CNV) burden. We combined data from 5,745 cases and 10,675 controls with other published datasets containing genome-wide CNV data. In this much-enlarged sample of 11,355 cases and 16,416 controls, we show for the first time that case CNVs are enriched for genes involved in GABAergic neurotransmission. Consistent with non-genetic reports of GABAergic deficits in schizophrenia, our findings now show disrupted GABAergic signaling is of direct causal relevance, rather than a secondary effect or due to confounding. Additionally, we independently replicate and greatly extend previous findings of CNV enrichment among genes involved in glutamatergic signaling. Given the strong functional links between the major inhibitory GABAergic and excitatory glutamatergic systems, our findings converge on a broad, coherent set of pathogenic processes, providing firm foundations for studies aimed at dissecting disease mechanisms.

## Highlights

• First genetic evidence for disruption of GABAergic signaling in schizophrenia
• No evidence for CNV disruption of biological processes beyond the CNS
• Support for involvement of NMDAR and ARC complexes in schizophrenia
• Additional, independent evidence for disruption of glutamatergic signaling

## Teaser

Pocklington et al. show for the first time that CNVs from individuals with schizophrenia are enriched for genes involved in GABAergic neurotransmission. Previous findings of CNV enrichment among genes involved in glutamatergic signaling are independently replicated and greatly extended.

## Introduction

Schizophrenia is a highly heritable disorder (Cardno and Gottesman, 2000), the genetic architecture of which includes a large number of alleles spanning the full spectrum of frequencies (Sullivan et al., 2012). It has been estimated that the additive effects of common variation, as indexed by alleles represented on the platforms used in genome-wide association studies (GWASs), contribute around a quarter to a third of the total population variance in schizophrenia liability. However, the 108 genome-wide-associated common variant loci reported in the largest GWAS study to date only explain a small fraction of this contribution (Schizophrenia Working Group of the Psychiatric Genomics Consortium, 2014). An increased burden of rare mutations has also been documented in schizophrenia, taking the form of both large CNVs (International Schizophrenia Consortium, 2008; Rees et al., 2014b; Walsh et al., 2008) and single-nucleotide variants (SNVs) (Purcell et al., 2014), which often occur as de novo mutations (Kirov et al., 2012; Malhotra et al., 2011; Xu et al., 2008). While several CNVs have been implicated in the disorder, no individual SNV has yet been robustly associated (Purcell et al., 2014). The CNVs (n = 11) strongly associated with schizophrenia in the largest systematic survey to date (Rees et al., 2014b) are in general large in both size (> 500 kb) and effect (ORs 2–60), the latter being in stark contrast with the small effects conferred by common alleles (typical OR < 1.1). Approximately 2.5% of patients and 0.9% of unaffected controls carry a CNV that is strongly supported as a risk factor for schizophrenia (Rees et al., 2014b). The pathogenic effects of these CNVs are not confined to schizophrenia; many increase risk for other disorders with a putative major neurodevelopmental component such as intellectual disability, autism spectrum disorder, and attention deficit hyperactivity disorder (Girirajan et al., 2012; Malhotra and Sebat, 2012; Williams et al., 2010).

A small number of single-gene CNVs have been associated with schizophrenia, but the only ones to be definitively implicated are deletions of *NRXN1* (Kirov et al., 2008; Rees et al., 2014b), which encodes the presynaptic cell adhesion protein neurexin 1. All other robustly associated CNVs span multiple genes making it difficult to infer the biological mechanism(s) through which they contribute to disease. Nevertheless, early pathway or gene set analyses of schizophrenia case-control CNV datasets indicated that case CNVs were enriched for synaptic and neurodevelopmental genes (Glessner et al., 2010; Walsh et al., 2008).

It has been noted that these initial approaches to pathway analysis did not completely control for confounds such as CNV size (Raychaudhuri et al., 2010). However, a study of parent-proband trios in which these factors were taken into account found that de novo CNVs in people with schizophrenia were enriched for synaptic proteins (Kirov et al., 2012). Moreover, this was largely the result of enrichment for genes encoding members of N-methyl-D-aspartate receptor (NMDAR) (Husi and Grant, 2001; Husi et al., 2000; Pocklington et al., 2006) and neuronal activity-regulated cytoskeleton-associated (ARC) protein complexes, both of which are known to be important for synaptic plasticity and cognitive function in rodents. When these same sets were additionally examined in large case-control datasets, case CNVs were found to contain an excess of genes from NMDAR, but not ARC, complexes (Kirov et al., 2012; Szatkiewicz et al., 2014). Exome sequencing studies have subsequently supported a role for both NMDAR and ARC complexes in disease (Fromer et al., 2014; Purcell et al., 2014). The same exome sequencing studies also found evidence of enrichment for rare disruptive and de novo point mutations among targets of fragile X mental retardation protein (FMRP) (Darnell et al., 2011), a finding that has also been reported for CNVs in a large schizophrenia case-control study (Szatkiewicz et al., 2014).

Here we present a detailed functional analysis of the largest schizophrenia CNV dataset for which full autosomal CNV data have been examined to date. The study is based on 11,355 cases and 16,416 controls from three separate studies: the International Schizophrenia Consortium (ISC), the Molecular Genetics of Schizophrenia (MGS), and a UK study of individuals diagnosed with schizophrenia and taking the anti-psychotic clozapine (CLOZUK) (International Schizophrenia Consortium, 2008; Levinson et al., 2011; Rees et al., 2014b). The ISC and MGS datasets were utilized in Kirov et al. (2012) to investigate CNV enrichment for ARC and NMDAR gene sets, while no CNV gene set analyses have yet been performed in CLOZUK. Starting from the hypothesis that schizophrenia reflects perturbation of brain function and development, our primary analysis focuses on a circumscribed set of annotations that are related to CNS function and development and are based on proteomic, RNA sequencing, and functional genetic data. In order to evaluate to what extent the pathogenic effects of CNVs primarily reflect disruption of brain function, as a secondary analysis we searched more widely for additional gene set enrichments using a more comprehensive range of annotations available from large, freely accessible databases.

## Results

We identified 134 gene sets relevant to various aspects of nervous system function and development, covering subcellular neuronal function, cellular physiology, cell morphology, brain region and fiber tract morphology, behavior, and brain development (Table S1). Gene sets were derived from functional studies of single genes recorded in the MGI Mammalian Phenotype (MP) database (Blake et al., 2014) with the exception of subcellular neuronal terms, which comprised a mixture of CNS-related gene sets taken from previous studies of schizophrenia (Fromer et al., 2014; Kirov et al., 2012; Purcell et al., 2014) as well as sets that were curated from the proteomic literature (see Table S1 for full list of references). To constrain multiple testing, we utilized a subset of the terms available from MGI (Table S1), which represented CNS annotations postulated to be of most likely relevance to schizophrenia, while at the same time retaining broad functional coverage to allow for the emergence of novel pathophysiological clues. Directional terms such as “decreased” or “enhanced” were avoided in favor of broader categories denoted by “abnormal,” “impaired,” etc.

### CNV Enrichment in Gene Sets with Strong Prior Evidence for Involvement in Schizophrenia

Consistent with recent approaches (Szatkiewicz et al., 2014), our analyses are based on large, rare CNVs (> 100 kb, frequency < 1%), as these are both the most robustly called and most enriched in people with schizophrenia. Gene set enrichment analysis was performed using a logistic regression model (Kirov et al., 2012) with covariates included to control for the size and total number of genes overlapping each CNV and for the source of the data (study and genotyping array used). As we were only interested in gene sets that were enriched for CNVs in cases, we used one-tailed tests.

Of the 134 CNS-related gene sets, we first evaluated those for which there existed prior, replicated evidence of enrichment for rare mutations in schizophrenia in at least three independent studies: the NMDAR protein network, ARC protein complex, and mRNA targets of FMRP. Here, as in all subsequent analyses, we first tested for enrichment in the combined set of CNVs and then deletions and duplications separately. The results of these analyses were Bonferroni corrected for the nine tests performed. NMDAR network genes were highly enriched in CNVs overall (P_corrected_ = 3.82 × 10^−8^), the signal primarily coming from duplications (P_corrected_ = 2.26 × 10^−8^). The ARC gene set was not enriched for CNVs overall, but was enriched in the secondary test of deletions (P_corrected_ = 0.0031), while FMRP targets displayed a modest trend toward enrichment in deletions (P_corrected_ = 0.076).

Both ISC and MGS samples were utilized in our previous study (Kirov et al., 2012) to investigate CNV enrichment for ARC and NMDAR. We therefore asked whether we would have found the above NMDAR and ARC association signals if we had performed our analysis in CLOZUK only. Restricting to CLOZUK samples, case CNVs were still enriched for both NMDAR network (P_corrected_ = 1.5 × 10^−6^ combined, P_corrected_ = 7.8 × 10^−7^ duplications) and ARC (P_corrected_ = 0.0077 deletions) gene sets. Thus, our analysis provides fully independent evidence for the NMDAR network and ARC complexes.

### Large CNVs Disrupt an Excess of CNS Gene Sets in Schizophrenia

We next investigated CNV enrichment for the full list of 134 CNS-related gene sets. To evaluate whether there was evidence for a general enrichment of CNS gene sets in our data, we tested whether the numbers of sets surpassing defined enrichment p value thresholds (P_uncorrected_ < 0.01, 0.001) were greater than expected. To do this, gene set enrichments for the full set of 134 CNS-related terms were compared to those generated from permuted data in which CNVs were randomly re-assigned among individuals, with the constraint that assignments were restricted to individuals from the same study so we could continue to allow for chip and study effects. In our analysis of deletions and duplications combined, more sets were enriched for CNVs in schizophrenia than expected under the null at all enrichment p value thresholds. This was also true when deletions and duplications were considered separately (Table 1).

To evaluate the significance of the tests of individual gene sets in a manner that allows easy comparison with our later analyses of the much larger annotation datasets (where permutation tests were computationally prohibitive), we adjusted gene set p values for multiple testing using Bonferroni correction for the 402 CNS gene set tests (134 sets × 3 analyses) performed (Table S1). Recognizing that this is over-conservative due to annotation overlap, in Table 2 we additionally list all gene sets with an uncorrected p < 0.001 under the combined test of all CNVs. As can be seen from Table 1, given the large excess in the observed number of associated sets compared with expectation (minimum N_obs_/N_exp_ = 50), most gene sets surpassing this threshold are likely to be true positives, even if they do not survive correction for multiple testing here. Functional processes captured by the six terms with a Bonferroni-corrected p value < 0.05 centered upon behavioral and physiological correlates of learning and related neuronal complexes.

For the combined analysis of all CNVs, after the NMDAR complex (P_corrected_ = 1.71 × 10^−6^) the next most highly associated term was the GABA_A_ receptor complex (P_corrected_ = 0.0012). Conditional analyses revealed these two signals to be essentially independent (see conditional analysis of GO and MGI below; see also Supplemental Experimental Procedures). Thus not only do we confirm, as noted above, the involvement of proteins involved in plasticity of the major excitatory system of the CNS, we also provide the first strong genetic evidence for an etiological role in the disorder for proteins affiliated with the major inhibitory system in the CNS, namely GABA_A_ receptor complexes (Heller et al., 2012).

### Deletions and Duplications Independently Enriched in CNS Gene Sets

Tables 3 and 4 list all gene sets with P_uncorrected_ < 0.001 for enrichment within deletions and duplications, respectively. After Bonferroni correction for the 402 CNS gene set tests, there were 14 terms with p value < 0.05 for enrichment in case deletions and 7 terms for case duplications. Enrichment for duplications was largely confined to behavioral and subcellular neuronal gene sets; terms associated via deletions extended over behavior, cellular physiology, subcellular complexes, and development. Deletions were most highly enriched for components of PSD-95 protein complexes (Fernández et al., 2009). PSD-95, a major postsynaptic scaffolding protein at glutamatergic synapses, interacts with a wide range of channels and receptors including NMDARs. It is notable that although the NMDAR and PSD-95 complexes are functionally related and have overlapping membership, the observations of strong (ORs > 3) and highly significant enrichments (both P_corrected_ < 10^−7^) for these sets relate to duplications and deletions, respectively. These findings are therefore based on sets of completely independent CNVs and, as such, provide extremely robust support for an etiological role for the disruption of glutamatergic signaling in schizophrenia.

### Disruption of CNS Gene Sets Extends beyond Known Schizophrenia Loci

Current data provide strong support for 11 CNV loci in schizophrenia: 6 deletions and 5 duplications (Rees et al., 2014b) (Table S2). Removing CNVs overlapping these known loci, we re-calculated CNS gene set enrichment. Deletion, duplication, and combined analyses all retained an excess of associated terms (Table 1); of the 14 gene sets enriched for deletions, 5 remained nominally associated (P_uncorrected_ < 0.05) when known loci were removed, as did 5 of the 7 terms enriched for duplications (Table S3).

### Individual Genes within Associated Gene Sets

To identify genes contributing most to gene set enrichment we calculated single gene association p values. This was done in the same manner as our gene set enrichment analyses, but with each “set” restricted to a single gene. For each CNS term with a Bonferroni-corrected p value < 0.05 we then extracted all genes with an uncorrected single gene p value < 0.05 (Tables S4, S5, and S6). To obtain significance at the level of an individual gene, there must be multiple observations of CNVs at the same region. It is therefore unsurprising that established recurrent CNV risk loci account for many such findings. Moreover, as recurrent CNVs are large, these frequently overlap multiple genes and contribute to multiple sets (e.g., del22q11; Table S5). It should be noted that some CNVs also hit multiple genes within a single set, but as each CNV only contributes once to the regression model (see Experimental Procedures), co-localization of set members does not inflate the significance of the set-based enrichment. A number of nominally associated genes lying outside established loci are well known to be important for neuronal signaling. These include the glutamate transporter *SLC1A1*, a recently reported candidate CNV locus for schizophrenia (Myles-Worsley et al., 2013; Rees et al., 2014a); GABAergic (*GABRD* also reported in Rees et al., 2014a) and nicotinic receptors (*CHRNA4*); synaptic scaffolding proteins *DLG2*, *DLGAP1*, and *SHANK2*; and key elements of the presynaptic vesicle release machinery *PCLO* and *NSF*.

Some of the nominally associated genes have been linked to Mendelian disorders with neurological symptoms, including Walker-Warburg syndrome, a congenital muscular dystrophy with brain and eye abnormalities (*ISPD* OMIM: 614643, *POMK* OMIM: 615249); nocturnal frontal lobe epilepsy type 1 (*CHRNA4* OMIM: 600513); generalized epilepsy (*GABRD* OMIM: 613060); spastic paraplegia 51, an autosomal recessive developmental disorder with severe intellectual disability (*AP4E1* OMIM: 613744); and Batten disease, an autosomal recessive neurodegenerative condition (*CLN3* OMIM: 204200).

### No Evidence for Gene Set Enrichment beyond CNS

We next determined whether any other gene sets had evidence for enrichment in case CNVs that was independent of the association signal captured by our primary CNS-related terms. For this we drew upon both the MGI MP database (Blake et al., 2014), from which we had derived most of our CNS-related gene sets, and the widely used Gene Ontology (GO) terms (Ashburner et al., 2000). As the MP database contains an extensive range of physiological, behavioral, and morphological phenotypes, but little of the low-level molecular function annotation present in GO, these two classifications are to an extent non-redundant and complementary.

We first identified a “minimal set” of terms capturing most of the enrichment signal arising from CNS-related gene sets. Taking the CNS terms surviving Bonferroni correction, we added the most significant term as a covariate to the regression model and recalculated gene set enrichment for each of the remaining terms. The term with the most significant residual enrichment was then added to the model, and the process repeated until there was no residual association (P_uncorrected_ < 0.05) in the remaining CNS annotations. Three terms were required to capture CNS gene set enrichment in the combined analysis of duplications and deletions: NMDAR network, GABA_A_ receptor complex, and abnormal behavior (see Supplemental Experimental Procedures). This indicates that there are independent enrichment signals in the NMDAR network and GABA_A_ receptor complex gene sets, as reported above. Conditioning on the above three CNS terms, no other GO or MP term survived Bonferroni correction (Tables S7 and S8).

CNS gene set enrichment for deletions was captured by three terms: PSD-95 complex, abnormal fear/anxiety-related behavior, and abnormal neural plate morphology (see Supplemental Experimental Procedures). Conditioning on these terms, no MP or GO term survived Bonferroni correction (Tables S7 and S8). Three terms captured CNS enrichment in duplications: associative learning, NMDAR network, and GABA_A_ receptor complexes (see Supplemental Experimental Procedures). Once again there was no evidence of additional gene set enrichment in either MP or GO annotations following Bonferroni correction (Tables S7 and S8).

### Pathogenicity of Large CNVs Is Related to the Number of CNS Genes Hit

Case CNVs > 100 kb were both larger (P_del_ = 6.8 × 10^−14^, P_dup_ = 0.37) and overlap (“hit”) a greater number of genes (P_del_ = 1.3 × 10^−16^, P_dup_ = 3.4 × 10^−5^) than those found in controls. However, even after conditioning on CNV size, the number of genes hit was strongly and independently associated (P_del_ = 1.0 × 10^−5^, P_dup_ = 2.9 × 10^−5^), whereas after conditioning on number of genes, the effect of size was much weaker and was restricted to deletions (P_del_ = 0.0073, P_dup_ = 0.49). The number of genes hit is therefore a better predictor of case-control status than CNV size.

We next investigated whether the relationship between number of genes hit and case-control status could be entirely attributed to genes within the disease-associated CNS annotations. To test this, we combined all the CNS annotations that had a Bonferroni-corrected p value < 0.05 to create a single associated CNS set (CNS_SZ_). We did this separately for deletions and duplications. The number of CNS_SZ_ genes hit by a CNV was a highly significant predictor of case-control status for both deletions and duplications (P_del_ = 1.1 × 10^−21^, P_dup_ = 1.7 × 10^−12^). Each was at least five orders of magnitude more significant than the corresponding analyses based on total number of genes hit (see previous paragraph). As expected this CNS_SZ_ term remained highly significant when conditioned on total number of genes hit in any category (P_del_ = 7.7 × 10^−7^, P_dup_ = 1.1 × 10^−9^), but the converse was not the case; conditioning on CNS_SZ_ there was little evidence of any remaining effect of the total number of genes hit by each CNV (P_del_ = 0.21, P_dup_ = 0.053).

### CNV Association Identifies Gene Sets Enriched for Rare, De Novo NS Mutations

Finally, we investigated whether associated CNS gene sets were also enriched in de novo non-synonymous (NS) mutations (Fromer et al., 2014). To constrain both the number and size of the gene sets tested, we collapsed the “minimal set” of terms capturing most of the CNS enrichment signal (see analysis of GO and MGI above) into a single gene set for each of our analyses (combined, deletion only, duplication only). Gene sets capturing the CNS enrichments for deletions and duplications were associated with de novo NS mutations observed in individuals with schizophrenia. This was not entirely due to ARC and NMDAR network genes (which we have previously described to be enriched for de novo NS mutations; Fromer et al., 2014) (Table 5). No enrichment was found when the analysis was repeated in a corresponding set of mutations from unaffected individuals (Table 5). When all 21 CNS terms with P_corrected_ < 0.05 (Tables 2–4) were tested individually, over half were nominally enriched for de novo NS mutations (cf. none for mutations from controls). While none survive correction for multiple testing (Table S9), this has to be interpreted in the context of the very weak enrichment in schizophrenia for de novo NS mutations, and therefore low power to robustly detect gene set enrichment. These findings independently support the broader relevance of the gene sets we have identified in the present study, but larger studies of de novo mutations will be required for finer-scale dissection.

## Discussion

**Figure 1:**
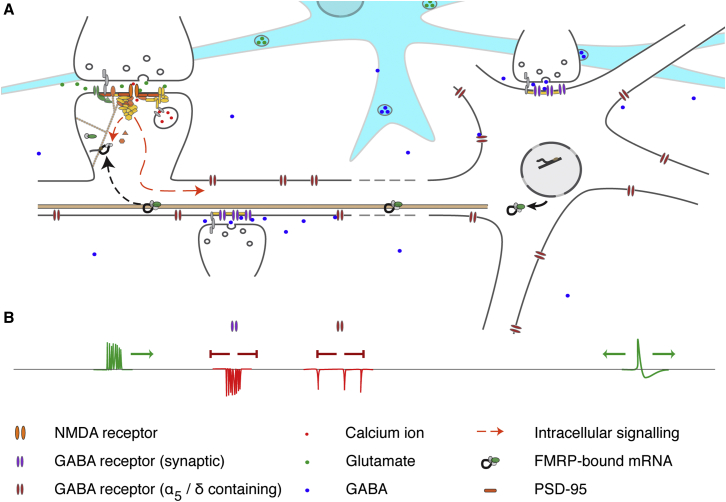
Functional Interactions between Neuronal Complexes Implicated in Schizophrenia Supporting and extending previous studies (Fromer et al., 2014; Kirov et al., 2012; Purcell et al., 2014), our analyses indicate a contribution to schizophrenia from ARC, NMDAR network, PSD-95, and GABA_A_ neuronal complexes. Although not strongly associated here, targets of the translational repressor FMRP have previously been found to be enriched in CNVs and rare de novo small mutations in individuals with schizophrenia (Fromer et al., 2014; Purcell et al., 2014; Szatkiewicz et al., 2014). This figure summarizes the relationship between these sets of molecules and their roles in synaptic signaling and plasticity. (A) PSD-95 complexes are an important component of the postsynaptic scaffold at glutamatergic synapses, linking a wide range of channels and receptors including NMDARs (top left). Calcium influx via the NMDAR drives multiple downstream pathways (red arrows): local signaling regulates induction of synaptic potentiation, while activation of ARC transcription via signaling to the nucleus is required for the long-term maintenance of synaptic changes. Once transcribed, mRNAs encoding ARC and other synaptic proteins are inactivated via association with FMRP and transported to synaptodendritic sites of protein synthesis. Here, activity-dependent dissociation of FMRP releases transcripts from translational repression allowing protein synthesis and incorporation into active synapses. (B) NMDAR activation requires both presynaptic glutamate release and strong post-synaptic depolarization, which may be induced by the back-propagation of action potentials. Influx of chloride ions via GABA receptors attenuates the dendritic transmission of excitation, inhibiting action potential generation and back-propagation. Phasic firing of synaptic GABA receptors plays a key role in establishing neural oscillations, required for the coordination of distributed functional networks. Tonic GABA receptors also modulate excitatory currents and oscillatory neuronal behavior, being responsive to local network activity via the overspill of GABA from synaptic receptors and its release/uptake by glia (blue cell in A). For simplicity all receptors are shown acting upon a single neuron; in reality, their interplay is distributed across multiple neuronal cell types, e.g., tonic GABA currents also modulating synaptic GABA release from interneurons.

We have performed a detailed, functionally informed analysis of large, rare CNVs from 11,355 schizophrenia cases and 16,416 controls. The results provide strong, novel evidence implicating disruption of inhibitory GABAergic modulation of neuronal signaling in schizophrenia and robustly confirm, and extend, the genetic evidence implicating disruption of excitatory glutamatergic signaling (Figure 1). It is clear, however, that these neuronal complexes do not entirely account for the enrichment of CNVs in cases given the independent enrichments seen in behavioral and neurodevelopmental gene sets. This suggests that subcellular processes beyond those currently ascribed to GABAergic and glutamatergic complexes remain to be identified.

We found no evidence that the pathogenic effects of CNVs reflect biological processes other than those directly relevant to brain function. This conclusion follows from the absence of additional gene set enrichments after conditioning on the CNS sets, and is further supported by the observation that the number of genes hit by a CNV in the disease-associated CNS pathways was a better predictor of whether a CNV occurred in a case or a control than total number of genes hit. This contrasts with recent findings based on common polymorphisms, where independent enrichments were found in enhancer elements that were active in both CNS and immune tissues (Schizophrenia Working Group of the Psychiatric Genomics Consortium, 2014), although a more recent analysis by that group suggests that most, if not all, of the signal is captured by the CNS enhancers (http://biorxiv.org/content/early/2015/01/23/014241).

While 11 CNV loci have been strongly associated with schizophrenia to date (Rees et al., 2014b), our results indicate that more associated CNVs remain to be identified. The association between number of genes hit and pathogenicity suggests that, in many instances, looking for a single gene explanation for CNV pathogenicity may not be fruitful. Instead it indicates that pathogenicity depends upon the total burden of relevant genes hit by a CNV, or that different single genes are implicated in different individuals depending upon their genetic and environmental context—the larger the CNV, the greater the probability that a critical pathway or process will be sufficiently impaired. It should be noted that the presence of multiple hits in the same CNV does not artificially inflate the significance of our enrichment tests, as each CNV only contributes once to the analysis.

There were strong, independent associations in postsynaptic complexes derived from glutamatergic synapses: NMDAR complex (Husi and Grant, 2001; Husi et al., 2000; Pocklington et al., 2006) genes were enriched in case duplications, while PSD-95 (Fernández et al., 2009) and to a lesser extent ARC complexes (Kirov et al., 2012) were enriched in deletions. When these findings are combined with existing evidence from de novo CNVs (Kirov et al., 2012), from rare SNVs and indels (Fromer et al., 2014; Purcell et al., 2014), and more recently from GWASs and common alleles (Schizophrenia Working Group of the Psychiatric Genomics Consortium, 2014), the relevance of altered glutamatergic signaling to schizophrenia etiology and pathophysiology seems to be beyond any reasonable doubt.

While models of schizophrenia based upon NMDAR hypofunction have a long history (Olney and Farber, 1995), the genetic data now indicate that the glutamatergic contribution to schizophrenia encompasses a much wider range of cellular processes converging upon synaptic information processing and plasticity (Figure 1). This is clearly inconsistent with hypotheses in which deficits in glutamatergic signaling primarily reflect disruption via neuromodulatory pathways (Stephan et al., 2009). Genetic evidence for disruption of neuromodulators is so far restricted to dopamine, with a genome-wide significant GWAS signal localized to *DRD2* (Schizophrenia Working Group of the Psychiatric Genomics Consortium, 2014). Both serotonergic 5-HT2_C_ and nicotinic α_7_ receptor complexes were tested here, and neither was found to be strongly associated (Table S1).

We also find novel, independent evidence for disruption of GABA_A_ receptor complexes (Heller et al., 2012) in schizophrenia. Deficits in GABAergic signaling have long been hypothesized to contribute to schizophrenia pathophysiology alongside perturbation of dopaminergic and glutamatergic systems (Carlsson, 1988; Lewis et al., 2012; Olney and Farber, 1995; Roberts, 1972). Evidence supporting a direct involvement of GABA has as yet not been compelling, being drawn from imaging studies and animal models of putative intermediate phenotypes, or post-mortem expression studies in small samples where reverse causality or confounding cannot be excluded (Inan et al., 2013). Here we find case CNVs to be enriched for components of GABA_A_ receptor complexes (Tables 2–4), with conditional analyses revealing the GABA_A_ association signal to be independent of that seen for NMDAR complex genes. Our results indicate that abnormalities in GABAergic signaling play a direct pathogenic role in schizophrenia and cannot be entirely attributed to secondary effects of NMDAR dysfunction (Laruelle et al., 2005; Lewis and Gonzalez-Burgos, 2006).

GABA_A_ receptor complex enrichment was strongest among duplications, where the most highly associated genes were α5, β3, and δ receptor subunits (Table S10). The genes encoding α5 and β3 subunits are found within the Angelman/Prader-Willi locus, while the δ subunit has been mapped to the critical region for the 1p36 deletion syndrome: a relatively common CNV associated with a range of neurodevelopmental outcomes (Battaglia et al., 2008; Shapira et al., 1997; Windpassinger et al., 2002) that has recently been identified as a candidate locus for schizophrenia (Rees et al., 2014a). The remaining autosomal GABA_A_ receptor genes largely cluster within two loci on chromosomes 4 and 5, neither of which displayed evidence of enrichment (Table S10). The GABA_A_ enrichment signal for deletions was driven by *NRXN1*, which encodes for the presynaptic cell adhesion protein neurexin 1, common to both GABAergic and glutamatergic synapses.

Multiple GABA_A_ receptor subtypes exist, each with a unique set of functional properties and a distinct spatiotemporal expression profile (reviewed by Fritschy and Panzanelli, 2014). In contrast to the β3 subunit, which is common to many receptor subtypes, α5 and δ subunits occur in distinct, mainly extrasynaptic populations of receptors responsible for tonic inhibition. This indicates that the contribution of GABAergic signaling to schizophrenia may not be primarily synaptic, although the presence of CNVs in *NRXN1* and *GHPN*, encoding neurexin 1 and the synaptic GABA receptor scaffolding protein gephyrin (Table S10), suggests that perturbation of synaptic GABAergic signaling may also play a role.

Tonic inhibition in the hippocampus, a process to which both α5- and δ-containing GABA_A_ receptors contribute (Glykys et al., 2008), alters the induction of long-term potentiation (Martin et al., 2010). Moreover, prolonged activation of NMDARs has been shown to reduce cell surface expression of δ-containing receptors (Joshi and Kapur, 2013). Thus, there are potential functional connections between our findings of enrichment for CNVs in GABAergic and postsynaptic glutamatergic complexes in schizophrenia. At the behavioral level, perturbation of NMDAR and tonic GABA signaling both lead to alterations in associative learning (Bauer et al., 2002; Martin et al., 2010; Rodrigues et al., 2001). This hierarchy of functionally related processes, from subcellular complexes to behavioral learning via cellular signaling and plasticity, encapsulates the elements of gene set enrichment common to case duplications and deletions (Tables 2–4).

Our findings are consistent with a considerable body of non-genetic literature; it is nearly 40 years since the cognitive deficits seen in schizophrenia were first proposed to reflect dysfunctional associative learning (Miller, 1976), with later hypotheses suggesting perturbed synaptic plasticity as the source of this dysfunction (Friston, 1998). Our identification of independent genetic associations in glutamatergic and GABAergic complexes is particularly relevant to proposals that alteration in the ratio of excitatory to inhibitory transmission (E/I balance) underlies the behavioral deficits seen in schizophrenia (reviewed by Kehrer et al., 2008; see also Yizhar et al., 2011). Discussions typically focus upon the oscillatory properties of neuronal networks, fundamental to efficient information transfer and the coordination of neuronal assemblies (Buzsáki and Draguhn, 2004; Buzsáki and Watson, 2012). Deficits in gamma rhythms have been reported in schizophrenia (reviewed by Uhlhaas and Singer, 2010), and while multiple mechanisms contribute to the generation of these rhythms (Bartos et al., 2007), deficits have primarily been hypothesized to result from the altered firing of GABAergic interneurons (Lewis and Gonzalez-Burgos, 2006; Uhlhaas and Singer, 2010). Interestingly, the frequency of hippocampal gamma oscillations is sensitive to the balance between NMDAR-dependent excitation and GABA δ subunit-dependent tonic inhibition of interneurons (Mann and Mody, 2010), linking these hypotheses directly to our findings.

Although less discussed in relation to schizophrenia, E/I balance also plays a role in the development and maintenance of stable perceptual and motor representations (reviewed by Carcea and Froemke, 2013). During early post-natal development unbalanced excitatory input drives activity-dependent plasticity, shaping emerging networks of synaptic connections in response to the environment. As networks mature and inhibitory elements are progressively integrated, E/I inputs become correlated making internal representations resistant to further modification. In the adult brain, activation of neuromodulatory systems (including acetylcholine, dopamine, and serotonin) can alter E/I balance, allowing sensory circuits to again become sensitive to environmental input. Unlike the experience-dependent modifications that occur during development, plasticity in adult sensory cortex is generally transient (see Carcea and Froemke, 2013). Genetic associations potentially link the main elements of these processes to schizophrenia: E/I signaling and synaptic plasticity through our analyses, dopaminergic signaling via the *DRD2* GWAS locus noted above (Schizophrenia Working Group of the Psychiatric Genomics Consortium, 2014). Disruption of such processes may potentially play a role in the developmental trajectory of schizophrenia, or in the manifestation of transient perceptual alterations during psychotic episodes.

In conclusion, our analyses support and extend previous studies (Fromer et al., 2014; Kirov et al., 2012; Purcell et al., 2014) indicating a contribution to schizophrenia from complexes central to the induction (NMDAR) and maintenance (ARC) of synaptic plasticity and provide strong novel evidence for the involvement of inhibitory modulation (GABA) of synaptic signaling (Figure 1). Perturbation of these processes is likely to have a widespread impact on brain function, and only a subset of genetic lesions within these systems may be compatible with a schizophrenia phenotype. The identification of the mechanisms by which disruption of these processes by genetic mutation leads to psychopathology will doubtless require experimental studies in model systems of high construct validity. The strength of genetic evidence converging on a plausible and coherent set of biological processes provides firm foundations upon which such studies can now proceed.

## Experimental Procedures

### Samples, Genotyping, and CNV Quality Control

Case and control CNVs were derived from three samples: CLOZUK, the ISC, and the MGS. A full description of these samples, the arrays they were genotyped on, and CNV calling procedures can be found in the Supplemental Experimental Procedures or in the original publications (International Schizophrenia Consortium, 2008; Levinson et al., 2011; Rees et al., 2014a). Briefly, CLOZUK samples were genotyped on several Illumina arrays. In order to limit any bias in detecting specific CNVs between the CLOZUK cases and controls, done on different arrays, for CNV calling we used only the 520,766 probes common to all these arrays. In the CLOZUK sample CNVs were called with PennCNV (Wang et al., 2007). All MGS samples were genotyped on Affymetrix 6.0 arrays, and approximately equal proportions of ISC cases and controls were genotyped on either Affymetrix 6.0 or Affymetrix 5.0 arrays (see Supplemental Experimental Procedures). In the MGS and ISC samples CNVs were detected using Birdsuite (Korn et al., 2008). Only samples with a European ancestry were retained for analysis. Rigorous quality control was performed to remove low-quality samples (full details presented in the Supplemental Experimental Procedures), resulting in 5,745 cases and 10,675 controls in CLOZUK, 2,214 cases and 2,556 controls in MGS, and 3,395 cases and 3,185 controls in ISC retained for analysis. Taking CNV calls from samples which passed quality control in each study, CNVs were joined if the distance separating them was less than 50% of their combined length. CNVs were excluded if they overlapped low copy repeats by more than 50% of their length, or had a probe density < 1 probe/20 kb. CNVs with a frequency > 1% or identified as false positives by an in silico median *Z* score outlier method were also removed (Kirov et al., 2012). *Z* score validation was not performed for the ISC study as we did not have access to the raw intensity data. Following QC, genes overlapping CNVs were identified using genomic locations for the appropriate build of the human genome: Build 35 of the human genome for ISC, Build 36 for MGS, and Build 37 for CLOZUK. Studies were then collated, and CNVs < 100 kb in size and/or covered by < 15 probes were removed prior to analysis. Differences in genotyping chip, CNV calling, and genome build between studies are controlled for in our enrichment analyses through the “chip” and “study” covariates; while between-study differences may reduce power to identify true positives, they do not increase the rate of false positives. See Supplemental Experimental Procedures for further details.

### Gene Annotations

Proteomic studies used to derive subcellular terms are listed in Table S1. For terms analyzed in Kirov et al. (2012), the processed gene sets analyzed in that study were re-used here. Gene sets for all other subcellular terms were extracted from the relevant studies and mapped to human coding genes. GO annotations were taken from NCBI gene2go (ftp://ftp.ncbi.nih.gov/DATA), using *Homo sapiens* annotations only. MP ontology and gene annotations were downloaded from the Mouse Genome Informatics (MGI) online resource (http://www.informatics.jax.org). Genes were mapped to human using the file HOM_MouseHumanSequence.rpt, also downloaded from MGI. For further details see Supplemental Experimental Procedures.

### Enrichment Test for Individual Gene Sets

For each gene set, the numbers of genes “hit” by case and control CNVs were compared; a gene was counted as being hit by a CNV if the CNV overlapped any part of its length. To overcome biases related to gene and CNV size, and to control for differences between studies and genotyping chips, the following logistic regression models were fitted to the combined set of CNVs:

(a) logit (pr(case)) = study + chip + CNV size + total number of genes hit
(b) logit (pr(case)) = study + chip + CNV size + total number of genes hit + number of genes hit in gene set

Comparing the change in deviance between models (a) and (b), a one-sided test for an excess of genes in the gene set being hit by case CNVs was performed. For further details and a full description of the approach taken for multiple testing correction (outlined in the main text), see Supplemental Experimental Procedures.

### Permutation Test for General Enrichment of CNS Gene Sets

Case-control status was permuted 1,000 times, status being shuffled between CNVs from the same study and genotyping chip (“Affymetrix 5.0,” “Affymetrix 6.0,” or “Illumina”). Enrichment analyses were performed in each permuted dataset; the proportion of datasets in which the number of terms with p < P_thr_ equaled or exceeded that of the true data being used as the empirical p value for an excess of associated terms at the threshold P_thr_.

### Enrichment beyond CNS-Related Terms

From CNS gene sets with P_corrected_ < 0.05, a subset was identified that captured the association signal in all other terms. GO and MGI terms were then analyzed using the enrichment test outlined above, but with this “minimal” set of terms added as covariates to the regression models (see main text and Supplemental Experimental Procedures).

### Removing Signal from Known Loci

To investigate whether gene set enrichment was solely driven by CNVs at loci well supported by current data (Table S2), we removed all CNVs overlapping these loci and re-ran the enrichment analysis as above. See Supplemental Experimental Procedures for further details.

### Single Gene Enrichment Analysis

This was performed in an identical manner to gene set enrichment analysis, but with each “gene set” here comprising a single gene. The term “number of genes hit in gene set” in model (b) thus becomes a binary variable.

### CNV Size and Number of Genes Hit as Predictors of Case-Control Status

CNV size and number of genes hit (either total or CNS_SZ_) were regressed against CNV case-control status under a logistic regression model. Covariates were included for study and genotyping chip (as in enrichment test, above). See Supplemental Experimental Procedures for further details.

### De Novo Rare Variant Analysis

NS de novo variants found in individuals with schizophrenia were taken from Fromer et al. (2014), consisting of variants identified in four separate studies (Fromer et al., 2014; Girard et al., 2011; Gulsuner et al., 2013; Xu et al., 2012). These were analyzed for gene set enrichment using the dnenrich software (Fromer et al., 2014) (http://bitbucket.org/statgen/dnenrich). NS de novo variants found in unaffected individuals were also taken from Fromer et al. (2014) and analyzed in an identical fashion. These consisted of healthy controls and unaffected siblings collated from six separate studies (Gulsuner et al., 2013; Iossifov et al., 2012; O’Roak et al., 2012; Rauch et al., 2012; Sanders et al., 2012; Xu et al., 2012).

## Author Contributions

A.J.P., M.C.O., and M.J.O. led the study and interpreted the findings. A.J.P. collated gene sets with the assistance of J.H., D.H.K., and P.H. A.J.P. designed and implemented the analytic approach, with P.H. providing guidance on correction for multiple testing. J.T.R.W. led recruitment of the CLOZUK sample. E.R. and G.K. were responsible for all CNV calling in all samples. K.D.C., J.L.M., and S.A.M. coordinated the genotyping of the CLOZUK sample. A.J.P., assisted by M.C.O. and M.J.O., wrote the first draft of the manuscript, which was then commented on by other authors.

## Figures and Tables

**Table 1:** Enrichment of CNS Gene Sets for Association Signal

	N_case_	N_ctrl_	Significance Threshold							
			0.01				0.001			
			N_exp_	N_obs_	p	P_adj_	N_exp_	N_obs_	p	P_adj_
All	8,139	10,469	1.3	23	< 0.001	< 0.006	0.2	13	< 0.001	< 0.006
Deletion	3,164	4,234	1.4	38	< 0.001	< 0.006	0.2	25	< 0.001	< 0.006
Duplication	4,975	6,235	1.4	14	0.004	0.024	0.2	10	0.001	0.006
All (minus known loci)	7,649	10,028	1.3	10	0.015	0.03	0.1	4	0.005	0.01
Deletion (minus known loci)	2,963	4,140	1.4	11	0.008	0.048	0.1	2	0.024	0.14
Duplication (minus known loci)	4,856	6,165	1.4	6	0.038	0.23	0.1	3	0.006	0.036

The number of CNS gene sets with association p value surpassing a pre-defined threshold (p < 0.01 or 0.001) was compared to that seen in permuted data (1,000 permutations of CNV case-control status). Columns list the number of case and control CNVs contributing to each analysis (N_case_ and N_ctrl_, respectively); the average number of gene sets with p value surpassing a given threshold in the permuted data, N_exp_; the actual number of gene sets surpassing the same threshold in the unpermuted data, N_obs_; the empirical probability of finding N_obs_ or more gene sets surpassing the p value threshold in the permuted data, p; and the Bonferroni-corrected probability, P_adj_. Results are given for the combined analysis of all CNVs and for the analysis of deletions and duplications separately; these are presented first for the full dataset and then for the subset of CNVs that do not overlap well-supported schizophrenia loci.

**Table 2:** Enriched CNS Gene Sets, Combined Analysis

	N_gene_	Combined			Deletion		Duplication	
		p	P_adj_	OR (95% CI)	p	P_adj_	p	P_adj_
NMDAR network	59	4.3×10^−9^	1.7×10^−6^	2.47 (1.8–3.44)	0.045	1	2.5×10^−9^	1.0×10^−6^
GABA_A_	15	3.0×10^−6^	0.0012	2.51 (1.65–3.97)	0.00068	0.27	5.4×10^−5^	0.022
Abnormal associative learning	193	1.6×10^−5^	0.0066	1.38 (1.19–1.61)	1.0	1	1.6×10^−10^	6.2×10^−8^
Abnormal long-term potentiation	145	2.0×10^−5^	0.0081	1.49 (1.24–1.8)	0.58	1	1.1×10^−6^	0.00044
Abnormal behavior	1,973	5.1×10^−5^	0.020	1.12 (1.06–1.19)	3.0×10^−6^	0.0012	0.05	1
Abnormal CNS synaptic transmission	371	5.5×10^−5^	0.022	1.22 (1.11–1.35)	5.1×10^−6^	0.002	0.12	1
Thin cerebral cortex	45	0.00018	0.071	1.91 (1.32–2.8)	0.12	1	0.0006	0.24
Abnormal consumption behavior	442	0.00019	0.077	1.24 (1.09–1.41)	0.059	1	0.0005	0.2
Abnormal cued conditioning behavior	68	0.00027	0.11	1.69 (1.24–2.35)	0.55	1	1.4×10^−5^	0.0055
Abnormal synaptic transmission	437	0.00027	0.11	1.18 (1.08–1.29)	1.1×10^−5^	0.0044	0.21	1
Abnormal learning/memory/conditioning	424	0.00031	0.12	1.18 (1.08–1.29)	7.3×10^−5^	0.029	0.089	1
PSD-95 (core)	58	0.00048	0.19	1.71 (1.28–2.28)	4.3×10^−11^	1.7×10^−8^	0.97	1
Abnormal contextual conditioning behavior	89	0.00061	0.24	1.53 (1.18–1.99)	0.52	1	0.00011	0.045

CNS gene sets with P_uncorrected_ < 0.001 in the combined analysis of deletions and duplications are listed along with the number of genes in each set, N_gene_; uncorrected (p) and Bonferroni-corrected (P_adj_) p values for enrichment in case CNVs; estimated odds ratios (OR); and p values for enrichment in case deletions and duplications when analyzed separately. Note that while the NMDAR network was analyzed prior to other terms in this table, here it is corrected for the same number of tests as other terms for ease of comparison. See also Tables S1 and S3.

**Table 3:** Enriched CNS Gene Sets, Deletions

	N_gene_	p	P_adj_	OR (95% CI)
PSD-95 (core)	58	4.3×10^−11^	1.7×10^−8^	4.62 (2.85–7.8)
Abnormal neural plate morphology	23	2.1×10^−7^	8.4×10^−5^	
Abnormal prepulse inhibition	74	3.3×10^−7^	0.00013	1.94 (1.46–2.76)
Abnormal behavior	1,973	3.0×10^−6^	0.0012	1.35 (1.2–1.54)
Abnormal fear/anxiety-related behavior	216	3.2×10^−6^	0.0013	1.74 (1.38–2.23)
Abnormal CNS synaptic transmission	371	5.1×10^−6^	0.002	1.56 (1.29–1.92)
Abnormal spatial working memory	38	5.6×10^−6^	0.0022	4.94 (2.33–14.56)
Abnormal synaptic transmission	437	1.1×10^−5^	0.0044	1.46 (1.23–1.74)
Abnormal emotion/affect behavior	369	1.1×10^−5^	0.0044	1.45 (1.23–1.75)
Abnormal neuron differentiation	206	2.8×10^−5^	0.011	2.51 (1.67–3.87)
Abnormal spatial learning	156	4.8×10^−5^	0.019	1.66 (1.3–2.12)
Abnormal social/conspecific interaction	243	4.8×10^−5^	0.019	1.56 (1.26–1.97)
Abnormal learning/memory/conditioning	424	7.3×10^−5^	0.029	1.44 (1.21–1.73)
Abnormal miniature excitatory postsynaptic currents	62	0.0001	0.041	2.74 (1.57–4.95)
Cav2_channels	202	0.00017	0.068	1.85 (1.33–2.59)
Abnormal excitatory postsynaptic currents	69	0.00025	0.10	1.95 (1.31–2.93)
Abnormal axon extension	46	0.00027	0.11	5.68 (2.21–17.65)
Abnormal depression-related behavior	76	0.00033	0.13	3.69 (1.75–8.54)
ARC	25	0.00034	0.14	1.7 (1.24–2.33)
Abnormal excitatory postsynaptic potential	59	0.00067	0.27	4.2 (1.64–12.87)
GABA_A_	15	0.00068	0.27	2.43 (1.36–4.49)
Abnormal nervous system development	801	0.00073	0.29	1.43 (1.17–1.75)
Abnormal aggression-related behavior	63	0.00075	0.30	3.33 (1.64–7.24)
Abnormal response to novelty	152	0.00079	0.32	1.48 (1.18–1.87)
Abnormal sensory capabilities/reflexes/nociception	590	0.0008	0.32	1.39 (1.13–1.7)

CNS gene sets with P_uncorrected_ < 0.001 in the analysis of deletions are listed along with number of genes in each set, N_gene_; uncorrected (p) and Bonferroni-corrected (P_adj_) p values for enrichment in case CNVs; and estimated odds ratios (OR). See also Tables S1 and S3.

**Table 4:** Enriched CNS Gene Sets, Duplications

	N_gene_	p	P_adj_	OR (95% CI)
Abnormal associative learning	193	1.6×10^−10^	6.2×10^−8^	1.73 (1.46–2.08)
NMDAR network	59	2.5×10^−9^	1.0×10^−6^	3.09 (2.09–4.67)
Abnormal long-term potentiation	145	1.1×10^−6^	0.00044	1.65 (1.34–2.04)
Abnormal avoidance learning behavior	56	1.6×10^−6^	0.00066	1.89 (1.45–2.47)
Abnormal cued conditioning behavior	68	1.4×10^−5^	0.0055	2.02 (1.41–3)
GABA_A_	15	5.4×10^−5^	0.022	2.8 (1.56–5.67)
Abnormal contextual conditioning behavior	89	0.00011	0.045	1.68 (1.28–2.23)
Abnormal consumption behavior	442	0.00050	0.20	1.27 (1.1–1.46)
Abnormal temporal memory	108	0.00052	0.21	1.56 (1.2–2.04)
Thin cerebral cortex	45	0.00060	0.24	1.94 (1.3–2.92)

CNS gene sets with P_uncorrected_ < 0.001 in the analysis of duplications are listed along with number of genes in each set, N_gene_; uncorrected (p) and Bonferroni-corrected (P_adj_) p values for enrichment in case CNVs; and estimated odds ratios (OR). Note that while the NMDAR network was analyzed prior to other terms in this table, here it is corrected for the same number of tests as other terms for ease of comparison. See also Tables S1 and S3.

**Table 5:** “Minimal” CNS Gene Sets, Enrichment for NS De Novo Rare Variants

	N_gene_	De Novo SNV	N Mutation		p	P_adj_	Minus ARC/NMDAR	
			Observed	Expected			N_gene_	p
Combined	1,991	schizophrenia	110	96.63	0.084	0.24	1,930	0.25
Deletion	287		27	13.96	0.0014	0.0042	255	0.011
Duplication	249		24	12.04	0.0015	0.0045	191	0.026
Combined	1,991	control	64	60.45	0.33	1	1,930	0.28
Deletion	287		10	8.81	0.39	1	255	0.24
Duplication	249		6	7.53	0.76	1	191	0.68

Gene sets capturing CNS enrichment in combined, deletion, and duplication analyses were tested for enrichment with rare, non-synonymous de novo mutations from individuals with schizophrenia. Listed are number of genes in each gene set (N_gene_); number of variants found within these genes (Observed); number of variants expected (Expected); uncorrected and Bonferroni-corrected p values (p, P_adj_), where correction is for the three gene sets tested; plus p values following removal of ARC and NMDAR genes (Minus ARC/NMDAR). Analysis was then repeated for NS de novo rare variants identified in unaffected controls (same correction procedure). See also Table S9.

## Supplemental Information

Document S1. Table S3 and Supplemental Experimental Procedures

Table S1. CNS Gene Set Association, Related to Tables 1–4

Table S2. Known Schizophrenia Loci, Related to Tables 1–4

Table S4. Enriched CNS Gene Sets, Single-Gene Association, Combined, Related to Results

Table S5. Enriched CNS Gene Sets, Single-Gene Association, Deletions, Related to Results

Table S6. Enriched CNS Gene Sets, Single-Gene Association, Duplications, Related to Results

Table S7. MGI Gene Set Association, Conditional Analysis, Related to Results

Table S8. GO Gene Set Association, Conditional Analysis, Related to Results

Table S9. Associated CNS Gene Sets—Overlap with NS De Novo Rare Variants, Related to Table 5

Table S10. GABA_A_ Receptor Complex, Single-Gene Enrichment, Complete, Related to Discussion

Document S2. Article plus Supplemental Information
