# Supplementary material for: Novel Findings from CNVs Implicate Inhibitory and Excitatory Signaling Complexes in Schizophrenia
Source: Neuron. 2015 Jun 3;86(5):1203–14. doi: 10.1016/j.neuron.2015.04.022 (PMC4460187; doi:10.1016/j.neuron.2015.04.022)
Supplement: Document S1. Table S3 and Supplemental Experimental Procedures [file mmc1.pdf]

Neuron

Supplemental Information

Novel Findings from CNVs Implicate Inhibitory and Excitatory Signaling Complexes in Schizophrenia

Andrew J. Pocklington, Elliott Rees, James T.R. Walters, Jun Han, David H. Kavanagh, Kimberly D. Chambert, Peter Holmans, Jennifer L. Moran, Steven A. McCarroll, George Kirov, Michael C. O’Donovan, and Michael J. Owen

Supplemental Information

1. Supplemental Data

Table S1: CNS gene set association, Related to Tables 1-4

Table S2: Known schizophrenia loci, Related to Tables 1-4

Table S3: Enriched CNS gene-sets, known loci removed, Related to Tables 2-4

Table S4: Enriched CNS gene sets, single-gene association (combined), Related to Results

Table S5: Enriched CNS gene sets, single-gene association (deletions), Related to Results

Table S6: Enriched CNS gene sets, single-gene association (duplications), Related to Results

Table S7: MGI gene set association, conditional analysis, Related to Results

Table S8: GO gene set association, conditional analysis, Related to Results

Table S9: Associated CNS gene sets - overlap with NS de novo rare variants, Related to Table 5

Table S10: GABA$_A$ receptor complex, single-gene enrichment (complete), Related to Discussion

2. Supplemental Experimental Procedures

Samples, genotyping and CNV quality control

CLOZUK

Molecular Genetics of Schizophrenia (MGS)

International Schizophrenia Consortium (ISC)

Additional CNV QC for CLOZUK, ISC and MGS

Validation of CLOZUK

Gene annotations

Gene set enrichment test

Enrichment beyond CNS-related terms

Identification of 'minimal set' capturing association signal in enriched CNS terms

Removing signal from known loci

Calculation of gene set odds ratios

CNV size and number of genes hit as predictors of case-control status

Correction for multiple testing

3. Supplemental References

Supplemental Data

Legends

Table S1: CNS gene set association, Related to Tables 1-4

Association results for all 134 CNS gene sets tested in the combined analysis of deletions and duplications together and the analysis of deletions or duplications separately. Uncorrected (P) and Bonferroni corrected (P adjusted) one-sided p-values for enrichment in case CNVs are given, together with the source of the gene set and the number of autosomal genes in each set (N gene). As an additional test exploring the sensitivity of our results to CNV calling, we repeated our analysis of CNS-related gene sets restricting to CNVs > 500kb where we can expect very high concordance between chips. Of the 28 associations we report with a corrected P < 0.05, only 1 was not nominally associated in CNVs >500kb.

Table S2: Known schizophrenia loci, Related to Tables 1-4

Confirmed schizophrenia loci were taken from the largest systematic survey to date (Rees et al., 2014b). For each locus we list its position (Position (Mb, hg19)) together with the number (N) and percentage of individuals carrying the CNV (rate (%)) for cases and controls from

each of the three contributing studies. The total number/rate of these 11 loci in cases and controls is also given for each study. In this table only CNVs spanning an entire locus are counted; for analyses involving the removal of known loci, all CNVs overlapping these loci are removed (see Supplemental Experimental Procedures).

Table S3: Enriched CNS gene-sets, known loci removed, Related to Tables 2-4

Initial columns summarise the association data for CNS gene sets with Bonferroni corrected p-value < 0.05 in each of the three analyses: deletions and duplications being analysed together (Combined) or separately. The final two columns give the sign of the regression coefficient and uncorrected p-value for each gene set when CNVs overlapping well-supported schizophrenia loci were removed. Gene sets with $P_{uncorrected} < 0.05$ after removal of known loci are highlighted in bold.

Table S4: Enriched CNS gene sets, single-gene association (combined), Related to Results

For each CNS gene set with Bonferroni corrected p-value < 0.05 in the analysis of deletions and duplications combined, this table lists those genes with an uncorrected single-gene association p-value < 0.05 (again, in a combined analysis of deletions and duplications together). In addition to gene identifiers and chromosomal locations, the table lists the number of case and control CNVs that overlap the gene (N case and N ctrl respectively), raw and Bonferroni corrected p-values (P, P adjusted), and whether the gene is found in a well-supported schizophrenia CNV locus (known locus, locus type). Bonferroni correction is for the total number of single gene tests. Genes lying outside the boundaries of a known CNV locus, but whose association signal was clearly driven by that locus, were annotated as lying in 'xxx (extended)', where xxx is the corresponding locus.

Table S5: Enriched CNS gene sets, single-gene association (deletions), Related to Results

For each CNS gene set with Bonferroni corrected p-value < 0.05 in the analysis of deletions, this table lists those genes with an uncorrected single-gene association p-value < 0.05 (again, in the analysis of deletions alone). Columns are identical to those found in Table S4.

Table S6: Enriched CNS gene sets, single-gene association (duplications), Related to Results

For each CNS gene set with Bonferroni corrected p-value < 0.05 in the analysis of duplications, this table lists those genes with an uncorrected single-gene association p-value < 0.05 (again, in the analysis of duplications alone). Columns are identical to those found in Table S4.

Table S7: MGI gene set association, conditional analysis, Related to Results

Association results for all MGI gene sets tested in the combined analysis of deletions and duplications together and the analysis of deletions or duplications separately. Uncorrected (P) and Bonferroni corrected (P adjusted) one-sided conditional p-values for enrichment in case CNVs are given, together with the gene set name, id and number of autosomal genes (N gene).

Table S8: GO gene set association, conditional analysis, Related to Results

Association results for all GO gene sets tested in the combined analysis of deletions and duplications together and the analysis of deletions or duplications separately. Uncorrected (P) and Bonferroni corrected (P adjusted) one-sided conditional p-values for enrichment in case CNVs are given, together with the gene set name, id and number of autosomal genes in each set (N gene).

Table S9: Associated CNS gene sets - overlap with NS de novo rare variants, Related to Table 5

For each gene set with a Bonferroni corrected P < 0.05 identified by our analyses (Tables 2-4), we investigated enrichment for non-synonymous (NS) *de novo* rare variants from individuals with schizophrenia. Here we list the number of genes in each gene set (N gene); the number of de novo rare variants found within these genes (N observed); the number of variants expected (N expected); plus uncorrected (P) and Bonferroni corrected (P adjusted) p-values. For comparison, this analysis was then repeated using NS *de novo* rare variants identified in controls, with exactly the same correction procedure. See Experimental Procedures for the source of variants used.

Table S10: $GABA_A$ receptor complex, single-gene enrichment (complete), Related to Discussion

Single-gene CNV counts and enrichment p-values for all genes in the GABA receptor complex gene set. Genes found on the X chromosome, which was not analysed here, are listed for completeness. Columns are as given in Tables S4-S6.

Supplemental Tables

Table S3:

	gene set	N_{gene}	before removal coeff	before removal P	after removal coeff	after removal P
Combined	**NMDAR network**	**59**	**+**	**$4.3x10^{-9}$**	**+**	**$1.0x10^{-6}$**
	$GABA_A$	15	+	$3.0x10^{-6}$	+	0.075
	**abnormal associative learning**	**193**	**+**	**$1.6x10^{-5}$**	**+**	**0.0071**
	**abnormal long term potentiation**	**145**	**+**	**$2.0x10^{-5}$**	**+**	**0.031**
	**abnormal behavior**	**1973**	**+**	**$5.1x10^{-5}$**	**+**	**0.0025**
	**abnormal CNS synaptic transmission**	**371**	**+**	**$5.5x10^{-5}$**	**+**	**0.015**
Deletion	**PSD-95 (core)**	**58**	**+**	**$4.3x10^{-11}$**	**+**	**0.0022**
	**abnormal neural plate morphology**	**23**	**+**	**$2.1x10^{-7}$**	**+**	**0.0097**
	abnormal prepulse inhibition	74	+	$3.3x10^{-7}$	-	0.53
	**abnormal behavior**	**1973**	**+**	**$3.0x10^{-6}$**	**+**	**0.015**
	**abnormal fear/anxiety-related behavior**	**216**	**+**	**$3.2x10^{-6}$**	**+**	**0.012**
	abnormal CNS synaptic transmission	371	+	$5.1x10^{-6}$	+	0.29
	abnormal spatial working memory	38	+	$5.6x10^{-6}$	+	0.13
	abnormal synaptic transmission	437	+	$1.1x10^{-5}$	+	0.14
	abnormal emotion/affect behavior	369	+	$1.1x10^{-5}$	+	0.083
	**abnormal neuron differentiation**	**206**	**+**	**$2.8x10^{-5}$**	**+**	**0.042**
	abnormal spatial learning	156	+	$4.8x10^{-5}$	+	0.089
	abnormal social/conspecific interaction	243	+	$4.8x10^{-5}$	-	0.61
	abnormal learning/memory/conditioning	424	+	$7.3x10^{-5}$	+	0.33
	abnormal miniature excitatory postsynaptic currents	62	+	0.00010	+	0.091
Duplication	**abnormal associative learning**	**193**	**+**	**$1.6x10^{-10}$**	**+**	**0.0017**
	**NMDAR network**	**59**	**+**	**$2.5x10^{-9}$**	**+**	**0.00066**
	abnormal long term potentiation	145	+	$1.1x10^{-6}$	+	0.27
	abnormal avoidance learning behavior	56	+	$1.6x10^{-6}$	+	0.10
	**abnormal cued conditioning behavior**	**68**	**+**	**$1.4x10^{-5}$**	**+**	**0.00060**
	**$GABA_A$**	**15**	**+**	**$5.4x10^{-5}$**	**+**	**0.043**
	**abnormal contextual conditioning behavior**	**89**	**+**	**0.00011**	**+**	**0.016**

Supplemental Experimental Procedures

Samples, genotyping and CNV quality control

Tables listing genotyping chips, number of probes and number of samples post QC for each of the 3 studies used in this analysis are given below. The CLOZUK study drew together samples genotyped on a range of Illumina chips, control samples being chosen to ensure chips were as similar to those for cases as possible. Given that different Illumina chips were used in the CLOZUK sample, only probes present on all of these chips (N=520,766) were used to call CNVs, ensuring that all CNVs called on one chip were capable of being called on the others.

CLOZUK

A full description and ascertainment of the CLOZUK cases is given in (Rees, Walters et al. 2014, PMID: 24163246). Briefly, the case sample utilised here consists of patients taking clozapine. Blood was obtained from these patients through collaboration with Novartis, the manufacturer of a proprietary form of clozapine (Clozaril). These patients were aged 18-90 and had received a recorded diagnosis of treatment resistant schizophrenia. In the UK, treatment resistant schizophrenia implies a lack of satisfactory clinical improvement to adequate trials of at least two other antipsychotics. We excluded those with diagnoses other than treatment resistant schizophrenia and those prescribed clozapine for off-license indications. All cases were genotyped on either Illumina HumanOmniExpress-12v1 or Illumina HumanOmniExpressExome-8v.1 arrays.

The CLOZUK control sample has been described previously (Rees et al., 2014). This sample consisted of four non-psychiatric control datasets obtained from either the Database of Genotypes and Phenotypes (dbGaP) or the European Genome-Phenome Archive (EGA).

CLOZUK	Source (accession ID)	Array (N probes)	N common Illumina probes used to Call CNVs	N samples post QC, Europeans only
SZ Batch 1	Broad Institute	HumanOmniExpress-12v1 (730,525)	520,766	2,148
SZ Batch 2	Broad Institute	HumanOmniExpressExome-8v1 (951,117)	520,766	3,205
SZ Batch 3	Broad Institute	HumanOmniExpressExome-8v1 (951,117)	520,766	392
The Genetic Architecture of **Smoking** and Smoking Cessation	dbGaP (phs000404.v1.p1)	Illumina HumanOmni2.5 (2,443,179)	520,766	938
High Density SNP Association Analysis of **Melanoma**: Case-Control and Outcomes Investigation	dbGaP (phs000187.v1.p1)	Illumina HumanOmni1_Quad_v1-0-B (1,051,295)	520,766	2,955
Genetic Epidemiology of Refractive Error in the **KORA** Study	dbGaP (phs000303.v1.p1)	Illumina HumanOmni2.5 (2,443,179)	520,766	1,857
**WTCCC2** project samples from National Blood Donors (NBS) Cohort	EGA (EGAD00000000024)	Illumina 1.2M (1,238,733)	520,766	2,363
**WTCCC2** project samples from 1958 British Birth Cohort	EGA (EGAD00000000022)	Illumina 1.2M (1,238,733)	520,766	2,562

These four datasets were derived from a study on smoking and smoking cessation (dbGaP phs000404.v1.p1), melanoma (dbGaP phs000187.v1.p1), refractive error (dbGaP phs000303.v1.p1) and WTCCC2 (EGA EGAD00000000024 and EGAD00000000022), which combined amount to 12,080 samples before QC. These were genotyped on Illumina HumanOmni2.5, Illumina HumanOmni1_Quad_v1-0-B, Illumina HumanOmni2.5 and Illumina 1.2M arrays respectively (see table above).

Principal component analysis (PCA) was performed to derive the ancestries of the CLOZUK cases and controls by combining the data with Hapmap genotypes. Samples were stratified

into those from a European, African or ‘other’ origin. In this paper we only included those of European origin. Further details can be found in (Rees et al., 2014a).

Raw intensity data from each case/control dataset (listed in the table above) were independently processed and analysed to account for potential batch effects. The PennCNV (Wang et al., 2007) algorithm with GC correction was used to detect CNVs from the 520,766 probes common to all Illumina arrays used to genotype the CLOZUK sample. Samples were subjected to rigorous QC and excluded if for any one of the following metrics they represented an outlier in their source dataset: Log R ratio standard deviation, B-allele frequency drift, wave factor and total number of CNVs called per person.

Molecular Genetics of Schizophrenia (MGS)

Details of the MGS cohort have been described elsewhere (Levinson et al., 2011). Our CNV analysis and QC of this sample has also been described previously (Rees et al., 2014a). Briefly, the samples were genotyped at the Broad Institute, Cambridge, Massachusetts, using Affymetrix 6.0 genotyping arrays. All schizophrenic patients met DSM-IV criteria for schizophrenia or schizoaffective disorder. CNVs were called using the Birdsuite algorithm (Korn et al., 2008).

**MGS**	**Array (N probes)**	**N samples post QC, Europeans only**
SZ cases	Affymetrix 6.0 (1,854,910)	2,215
Controls	Affymetrix 6.0 (1,854,910)	2,556

International Schizophrenia Consortium (ISC)

Details of the ISC sample have been described elsewhere (International Schizophrenia Consortium, 2008). The sample was genotyped at the Broad Institute, Cambridge,

Massachusetts, using Affymetrix 6.0 or Affymetrix 5.0 genotyping arrays and consists of six European populations. CNVs were called using the Birdsuite algorithm (Korn et al., 2008).

**ISC**	**Array (N probes)**	**N samples post QC, Europeans only**
SZ cases	Affymetrix 6.0 (1,854,910)	1,583
Controls	Affymetrix 6.0 (1,854,910)	2,095
SZ cases	Affymetrix 5.0 (440,638)	1,812
Controls	Affymetrix 5.0 (440,638)	1,090

Additional CNV QC for CLOZUK, ISC and MGS

For individuals/CNVs passing QC procedures performed by the original studies, CNV calls were joined if the distance separating them was less than 50% of their combined length. CNV calls were excluded if they overlapped with low copy repeats by more than 50% of their length, or had a probe density (calculated by dividing the size of the CNV by the number of probes covering it) less than 1 probe/20kb. We used PLINK (Purcell et al., 2007) to remove CNVs with a frequency > 1% in their respective sample (CLOZUK, ISC or MGS). We then applied an *in silico* median Z-score outlier method of CNV validation, described in detail elsewhere (Kirov et al., 2012; Rees et al., 2014), to all remaining CNVs. This method has been shown to be effective for the removal of false positive CNV calls and detecting CNVs missed by calling (Kirov et al., 2012). We did not perform Z-score validation for the ISC study as we did not have access to the raw intensity data.

Following QC (performed separately for each study), protein-coding genes overlapping CNVs were identified using genomic locations for the appropriate build of the human genome: Build 35 of the human genome for ISC, Build 36 for MGS and Build 37 for CLOZUK. Studies were then collated and CNVs <100kb in size and/or covered by < 15 probes removed prior to analysis. Non-European individuals were removed prior to analysis, leaving 5,745 cases and 10,675 controls in CLOZUK; 2,215 cases and 2,556 controls in

MGS; and 3,395 cases and 3,185 controls in ISC. The number of CLOZUK cases used in the current study differs from that reported in (Rees et al., 2014a) as that study included an additional 571 cases from the CardiffCOGs sample.

In performing gene set enrichment analyses we specifically included covariates for genotyping chip and study to remove any biases due to differences between Affymetrix 5.0, Affymetrix 6.0 and Illumina arrays and between the cohorts used in each individual study. We would add that since matched sets of cases and controls were genotyped on each Affymetrix array, the use of multiple chips in ISC and MGS does not cause an increase in false positives. To investigate whether batch effects in CLOZUK (due to the multiple sources of controls) were driving our results, we took all CNS-related gene sets with a Bonferroni corrected p-value < 0.05 and tested for significant differences in CNV overlap between controls genotyped in different studies or on different chips. This was performed using the same logistic regression model as the case-control CNV enrichment test from our primary analysis, but with 'cases' now being control CNVs from one study/chip and 'controls' being control CNVs from a different chip/study (and obviously using no covariates for chip or study). Calculating two-sided p-values for all potential chip-chip and control study-control study pairings, there were no significant differences after correcting for the number of comparisons made (data not shown).

Validation of CLOZUK:

Validation of Clinical Diagnosis

We used the Cardiff Cognition in Schizophrenia (Cardiff COGS) sample to assess the validity of a psychiatrist-assigned diagnosis of treatment resistant schizophrenia as applied in CLOZUK. The Cardiff COGS sample is a conventional sample of those with schizophrenia

recruited via secondary care, mainly outpatient, mental health services in Wales and England. The recruitment procedures included inviting patients from clozapine clinics, irrespective of diagnosis. Consenting participants were interviewed with the Schedules for Clinical Assessment in Neuropsychiatry (SCAN) (Wing et al., 1990) and consensus research diagnoses were agreed with reference to the interview and clinical notes according to DSM-IV criteria.

Validation Procedure

Prior to the research interview we obtained clinicians' diagnoses for all participants in Cardiff COGS. From participants on clozapine we selected those with a clinical diagnosis of schizophrenia and confirmed that this matched the diagnosis provided when the participant was started on clozapine (i.e. treatment resistant schizophrenia) so as to be equivalent to the samples identified as having schizophrenia in CLOZUK. We then compared this diagnosis with the consensus research DSM-IV diagnosis.

Results

We identified 214 participants within CardiffCOGS (n=905) who were taking clozapine and had a clinician-assigned diagnosis of treatment resistant schizophrenia. Following consensus research diagnosis, 194 of these participants were identified as having DSMIV schizophrenia or schizoaffective disorder depressed sub-type, giving a positive predictive value (PPV) of 90.7%.

Many international groups and consortia also consider other diagnoses as 'schizophrenia' samples, namely schizoaffective disorder bipolar type, delusional disorder and schizophreniform disorders (Schizophrenia Working Group of the Psychiatric Genomics Consortium, 2014). If we expand our analysis to include these categories then 210 of 214

(PPV=98.1%) of those on clozapine with a clinical diagnosis of schizophrenia would receive a DSMIV research diagnosis of one of these schizophrenia spectrum disorders.

These results are entirely consistent with equivalent reports of the validity of clinician diagnoses in two Scandanavian studies (Ekholm et al., 2005; Jakobsen et al., 2005).

Molecular/Genetic Validation

In the largest GWAS meta-analysis to date, the schizophrenia working group of the Psychiatric Genomics Consortium identified 40 target subgroups within their primary GWAS analysis and performed a leave-one-out analysis (Schizophrenia Working Group of the Psychiatric Genomics Consortium, 2014). Using risk alleles identified in the remainder of the primary sample, polygenic risk profile scores were calculated for all individuals in the target subgroup; the ability of these scores to distinguish between cases and controls was then evaluated. The predictive value of the risk profile score when applied to CLOZUK was indistinguishable from its performance in other schizophrenia subgroups, indeed the values for R2 (on the liability scale) for CLOZUK are the 5th highest of all subsamples, implying that CLOZUK is one of the samples most highly enriched for schizophrenia risk alleles (see data for 'noclo_clo' in Extended data Figure 6b from (Schizophrenia Working Group of the Psychiatric Genomics Consortium, 2014)). In terms of CNVs, the rate of individual confirmed schizophrenia loci in CLOZUK are entirely consistent with those of the other schizophrenia studies (Table S2 of this paper).

Taken together, the clinical and molecular evidence strongly validate CLOZUK as a schizophrenia sample.

Gene annotations

The ARC and NMDAR network gene sets used here were taken from (Kirov et al., 2012); the $GABA_A$ receptor complex gene set is listed in Table S10. All other gene sets are available from the authors upon request.

GO

Gene Ontology (GO) annotations were taken from NCBI gene2go (ftp://ftp.ncbi.nih.gov/DATA), using Homo Sapiens annotations only. Parent terms were identified for each GO term through the AmiGO ontology (http://www.geneontology.org/GO.downloads.ontology.shtml). We used "is_a" and "part_of" (but not "regulates") to define child-parent relationships between terms. The parent terms of each GO term assigned to a gene in gene2go were also assigned to that gene. When performing enrichment analyses we restricted to GO terms containing between 20 and 2000 autosomal genes, a total of 4026 terms.

MGI

The Mammalian Phenotype (MP) ontology and gene annotations were downloaded from the Mouse Genome Database (Blake et al., 2011) within the Mouse Genome Informatics (MGI) online resource (http://www.informatics.jax.org). Gene annotations arising from transgene and multi-gene manipulations were removed. Parent terms were identified for each MP term and assigned to all genes annotated with that child term. Genes were mapped to human using file HOM_MouseHumanSequence.rpt, also downloaded from MGI. Within this file human and mouse genes are organised into orthologous groups identified by HomoloGene id. To ensure the unambiguous annotation of human genes, we discarded all phenotypic information from mouse genes with non-unique (1-many, many-1, many-many) orthology relationships (i.e. HomoloGene groups containing multiple mouse and/or human gene ids).

When performing enrichment analyses we restricted to MGI terms with more than 20 autosomal genes, a total of 2616 terms (of which 118 were extracted for use as CNS-related gene sets). As MGI terms relate to specific biological processes we felt there was no need to place an upper bound on gene set size, used above to remove extremely large, generic GO annotations.

Gene set enrichment test

For each gene set, the number of genes 'hit' by case and control CNVs were compared; a gene was counted as being hit by a CNV if the CNV overlapped any part of its length. To overcome biases related to gene and CNV size, and to control for differences between studies and genotyping chips, the following logistic regression models were fitted to the combined set of CNVs:

(a) logit (pr(case)) = study + chip + CNV size + total number of genes hit

(b) logit (pr(case)) = study + chip + CNV size + total number of genes hit + number of genes hit in gene set

Comparing the change in deviance between models (a) and (b), a one-sided test for an excess of genes in the gene set being hit by case CNVs was performed.

By comparing case to control CNVs, this analysis allows for the possibility of non-random CNV location unrelated to disease (i.e. CNVs tend to occur in specific locations of the genome and this is unrelated to case status, both in cases and controls). The inclusion of CNV size in the regression allows for the fact that case CNVs are larger than control CNVs

(and thus likely to hit more genes, regardless of function), even when restricting to those >100kb in length (see Results). Inclusion of the total number of genes hit in the regression corrects for case CNVs hitting more genes overall (regardless of function) than control CNVs. It should also be noted that since we compare between cases and controls, gene size (which is the same in cases and controls) is not a source of potential bias: CNVs of given size have exactly the same chance of overlapping a particular gene in both cases and controls.

Since case and control samples from the CLOZUK study were genotyped on different chips, we were unable to completely control for possible inter-chip differences. This is unlikely to influence our analyses: calling is most robust for large CNVs; calling was restricted to probes present on all arrays; and the arrays used were in any case comparable in coverage (Rees et al., 2014a). The chip covariate therefore took the values 'Affymetrix 5.0' (subset of ISC samples), 'Affymetrix 6.0' (subset of ISC and all MGS samples) and 'Illumina' (all CLOZUK samples). As a further check we took all CNS-related gene sets with a Bonferroni corrected p-value < 0.05 and tested for differences in CNV overlap between controls genotyped in different studies or on different chips (see 'Samples, genotyping and CNV quality control: Additional QC' above); no significant differences were found.

Enrichment beyond CNS-related terms

To determine whether any GO or MGI annotation showed evidence for enrichment in case CNVs that was independent of the association signal captured by CNS-related gene sets, the following regression models were fitted.

(a) logit (pr(case)) = study + chip + CNV size + total number of genes hit + CNS terms

(b) logit (pr(case)) = study + chip + CNV size + total number of genes hit + CNS terms + number of genes hit in gene set (from GO or MGI)

where CNS terms = number of genes hit in CNS gene set X + number of genes hit in CNS gene set Y + …

These were constructed by adding a subset of CNS terms, capturing the enrichment signal arising from all CNS gene sets with $P_{corrected} < 0.05$, to the regression models described earlier (see 'Enrichment test' above). The identification of this CNS subset is described in the main text and in greater detail below, its sole purpose being to minimise the number of additional model parameters to be fitted (i.e. compared to adding all CNS terms with $P_{corrected} < 0.05$). Comparing the change in deviance between models (a) and (b), a one-sided test for an excess of genes in the GO or MGI gene set being hit by case CNVs was performed.

Identification of 'minimal set' capturing association signal in enriched CNS terms

To capture the enrichment signal arising from CNS gene sets with $P_{corrected} < 0.05$, we added the most significant such term as a covariate to the regression model and recalculated gene set enrichment for each of the remaining terms. The term with the most significant residual enrichment was then added to the model and the process repeated until there was no residual association ($P_{uncorrected} < 0.05$) in the remaining terms. This iterative procedure is captured in the tables below. Initial columns (up to P) summarise the association data for CNS gene sets with Bonferroni $P_{corrected} < 0.05$. The remaining columns identify terms successively added to the original regression model and list residual enrichment p-values for the resulting extended model. The most significant p-value at each stage of the analysis (identifying the next term to be added to the model) is highlighted in bold.

For example, in the combined analysis of deletions and duplications the most significantly associated gene set was the NMDAR network gene set (see column 'P' in 'Combined' table below). With this is included as an extra covariate, the original regression model now becomes:

(a) logit (pr(case)) = study + chip + CNV size + total number of genes hit + number of NMDAR genes hit

To find the residual enrichment of the remaining gene sets (column 'NMDAR network' in 'Combined' table below), we compare the change in deviance between models (a) and (b):

(b) logit (pr(case)) = study + chip + CNV size + total number of genes hit + number of NMDAR genes hit **+ number of genes hit in gene set**

performing a one-sided test for an excess of genes in the gene set being hit by case CNVs. The $GABA_A$ gene set, which has the most significant residual association, is then added to (a) and the process repeated until no term has a residual $P_{uncorrected} < 0.05$ (see final column in 'Combined' table below).

Combined:

	N_{gene}	P	NMDAR network	$GABA_A$	abnormal behavior
NMDAR network	59	**4.3×10^{-9}**	1	1	1
$GABA_A$	15	3.0×10^{-6}	**7.1×10^{-6}**	1	1
abnormal associative learning	193	1.6×10^{-5}	0.0060	0.028	0.088
abnormal long term potentiation	145	2.0×10^{-5}	0.0054	0.020	0.091
abnormal behavior	1973	5.1×10^{-5}	0.00032	**0.0052**	1
abnormal CNS synaptic transmission	371	5.5×10^{-5}	0.0016	0.020	0.24

Deletion:

	N_{gene}	P	PSD-95 (core)	abnormal fear/anxiety-related behavior	abnormal neural plate morphology
PSD-95 (core)	58	**$4.3x10^{-11}$**	1	1	1
abnormal neural plate morphology	23	$2.1x10^{-7}$	0.00015	**0.0020**	1
abnormal prepulse inhibition	74	$3.3x10^{-7}$	0.034	0.27	0.67
abnormal behavior	1973	$3.0x10^{-6}$	0.013	0.40	0.44
abnormal fear/anxiety-related behavior	216	$3.2x10^{-6}$	**0.00015**	1	1
abnormal CNS synaptic transmission	371	$5.1x10^{-6}$	0.090	0.50	0.67
abnormal spatial working memory	38	$5.6x10^{-6}$	0.0029	0.043	0.18
abnormal synaptic transmission	437	$1.1x10^{-5}$	0.060	0.79	0.85
abnormal emotion/affect behavior	369	$1.1x10^{-5}$	0.0016	0.72	0.94
abnormal neuron differentiation	206	$2.8x10^{-5}$	0.0046	0.026	0.052
abnormal spatial learning	156	$4.8x10^{-5}$	0.0018	0.26	0.35
abnormal social/conspecific interaction	243	$4.8x10^{-5}$	0.16	0.66	0.89
abnormal learning/memory/conditioning	424	$7.3x10^{-5}$	0.055	0.90	0.93
abnormal miniature excitatory postsynaptic currents	62	0.00010	0.58	0.45	0.36

Duplication:

	N_{gene}	P	abnormal associative learning	NMDAR network	$GABA_A$
abnormal associative learning	193	**$1.6x10^{-10}$**	1	1	1
NMDAR network	59	$2.5x10^{-9}$	**$2.7x10^{-5}$**	1	1
abnormal long term potentiation	145	$1.1x10^{-6}$	0.15	0.33	0.42
abnormal avoidance learning behavior	56	$1.6x10^{-6}$	0.18	0.38	0.20
abnormal cued conditioning behavior	68	$1.4x10^{-5}$	0.20	0.12	0.25
$GABA_A$	15	$5.4x10^{-5}$	0.0051	**0.0047**	1
abnormal contextual conditioning behavior	89	0.00011	0.69	0.47	0.75

Removing signal from known loci

To investigate whether gene set enrichment was solely driven by CNVs at loci well supported by current data, we removed all CNVs overlapping these loci and re-ran the enrichment analysis. To identify CNVs for removal, we collated a list of all genes lying in known CNV loci, plus any neighbouring genes whose association signal was also clearly driven by these loci. CNVs hitting one or more of these genes were then removed prior to re-analysis. When analysing deletions all known deletion loci were removed; when analysing duplications all known duplication loci were removed; and when analysing deletions and duplications combined, all CNVs overlapping a known locus were removed irrespective of their class (deletion/duplication).

Calculation of gene set odds ratios

In order to calculate odds ratios for enriched gene sets, the following logistic regression model was fitted to the full set of individuals from each study (i.e. including those in which no large CNVs were identified):

logit (pr(case)) = study + average CNV size + number of CNVs + total number of genes hit + number of genes hit in gene set

where 'average CNV size' is the mean length of all CNVs >100kb for that individual; 'number of CNVs' is the total number of CNVs > 100kb for that individual; 'total number of genes hit' and 'number of genes hit in gene set' count the corresponding number of unique genes hit by these CNVs (any gene hit by two CNVs would only count once). The odds ratio was derived from the coefficient of the 'number of genes hit in gene set' term. Since the unit of analysis is now the individual rather than the CNV, we control for average CNV length and CNV number in line with the recommendations of (Raychaudhuri et al., 2010).

CNV size and number of genes hit as predictors of case-control status

When investigating the relationship between CNV size, number of genes hit and case-control status, the following four models were fitted:

(a) logit (pr(case)) = study + chip

(b) logit (pr(case)) = study + chip + CNV size

(c) logit (pr(case)) = study + chip + total number of genes hit

(d) logit (pr(case)) = study + chip + CNV size + total number of genes hit

Comparing the change in deviance between models (a) and (b), a two-sided test was used to assess the relationship between CNV size and case-control status; likewise, a comparison between (a) and (c) was made for total number of genes hit. Comparison between (c) and (d) was used to assess the relationship between CNV size and case-control status conditional on total number of genes hit, comparison between (b) and (d) giving the analogous result for total number of genes hit conditional on CNV size.

Genes from all CNS annotations with a Bonferroni corrected p-value < 0.05 were combined to create a single associated CNS set (CNS_{SZ}). One such set was created for deletions, another for duplications. A comparison between total number of genes hit and number of CNS_{SZ} genes hit was also performed, with 'number of CNS_{SZ} genes hit' replacing 'CNV size' in the above regression models. Very similar results were obtained when CNS_{SZ} was constructed using only the much smaller ‘minimal’ subsets of annotations (see above) that capture the bulk of CNS enrichment (data not shown).

Correction for multiple testing

Analyses fall into two main classes, 1) gene set enrichment tests to identify significant associations and 2) subsequent ancillary analyses to investigate the source of any notable enrichment.

1) These comprised primary tests of previously associated gene sets (ARC, NMDAR and FMRP); secondary tests of CNS-related gene sets; and finally tertiary tests of the more comprehensive GO and MGI annotations. At each stage, analyses were performed first for

the combined CNV sample and then for deletions and duplications separately. At each stage of our analysis, gene set enrichment p-values were Bonferroni corrected for the total number of tests performed up to that point, as listed in the table below.

Gene sets	CNV tests	N test (novel)	N test (total)
ARC, NMDAR network, FMRP	Combined, Deletion & Duplication	9	9
CNS-related	Combined, Deletion & Duplication	393	402
MGI (2498 terms) + GO (4026 terms)	Combined, Deletion & Duplication	19572	19974

To test for enrichment with rare, non-synonymous *de novo* mutations from individuals with schizophrenia, the 'minimal set' of terms that capture most of the CNS enrichment signal were collapsed into a single gene set for each of our analyses (combined, deletion and duplication). Results were Bonferroni corrected for these 3 tests. An ancillary analysis was then performed to investigate whether the association signals identified were solely due to ARC and NMDAR genes. As we only explore the source of enrichment signals and do not claim to find novel associations, p-values for these tests are uncorrected. To check that enrichment was not due to some property of NS variants unrelated to disease, the analysis was then repeated using NS *de novo* rare variants identified in unaffected individuals. Results were again corrected for 3 tests. Analyses of the 21 individual gene sets listed in Table S9 were Bonferroni corrected for 3 + 21 = 24 tests.

2) Since ISC and MGS data had previously been used to investigate CNV enrichment for ARC and NMDAR (Kirov et al., 2012), we were interested in investigating whether the enrichment seen in the present combined ISC-MGS-CLOZUK sample was solely due to ISC and MGS. As we are simply investigating the partitioning of the association signal between datasets, it does not make sense to correct for tests performed in the full sample. CLOZUK-only results for the combined CNV analysis and for the analysis of deletions and duplications

separately were corrected for 9 tests.

Prior to discussing CNV enrichment for individual CNS gene sets, we investigate whether the 134 sets as a whole display more evidence of nominal association than would be expected by chance, performing permutation tests at two p-value thresholds separated by an order of magnitude (0.01 and 0.001) (see Table 1). Results are corrected for 6 tests, corresponding to the 2 thresholds x 3 analyses (combined, deletion only and duplication only).

To quantify the effect of removing known loci we employed a permutation test in exactly the same manner, results being given in the lower half of (Table 1). The correction procedure here is identical.

To identify genes contributing most to gene set enrichment we calculated single gene association p-values, listing genes with uncorrected $P < 0.05$ in Tables S4-S6. The number of genes tested in each analysis were: 10200 for the combined analysis, 3918 for deletions and 8759 for duplications, these being the number of genes overlapping at least one contributing CNV. In these tables, single gene enrichment p-values are corrected for the full 10200 + 3918 + 8759 = 22877 single gene tests.

The section investigating correlation between case-control status and CNV size and number of genes disrupted falls outside the two main classes of analysis discussed above. The initial analysis of size and number of genes is corrected for 4 tests (size and number in deletions and duplications), the subsequent 2 tests for CNS_{SZ} are corrected for the full set of 6 tests. Conditional analyses, in which we only explore the source of enrichment signals and do not claim to find novel associations, remain uncorrected.

Supplemental References

Blake, J.A., Bult, C.J., Kadin, J.A., Richardson, J.E., and Eppig, J.T. (2011). The Mouse Genome Database (MGD): premier model organism resource for mammalian genomics and genetics. Nucleic Acids Res 39, D842-848.

Ekholm, B., Ekholm, A., Adolfsson, R., Vares, M., Osby, U., Sedvall, G.C., and Jonsson, E.G. (2005). Evaluation of diagnostic procedures in Swedish patients with schizophrenia and related psychoses. Nordic journal of psychiatry 59, 457-464.

Jakobsen, K.D., Frederiksen, J.N., Hansen, T., Jansson, L.B., Parnas, J., and Werge, T. (2005). Reliability of clinical ICD-10 schizophrenia diagnoses. Nordic journal of psychiatry 59, 209-212.

Purcell, S., Neale, B., Todd-Brown, K., Thomas, L., Ferreira, M.A., Bender, D., Maller, J., Sklar, P., de Bakker, P.I., Daly, M.J., and Sham, P.C. (2007). PLINK: a tool set for whole-genome association and population-based linkage analyses. Am J Hum Genet 81, 559-575.

Wing, J.K., Babor, T., Brugha, T., Burke, J., Cooper, J.E., Giel, R., Jablenski, A., Regier, D., and Sartorius, N. (1990). SCAN. Schedules for Clinical Assessment in Neuropsychiatry. Arch Gen Psychiatry 47, 589-593.
