# Supplementary material for: Novel Findings from CNVs Implicate Inhibitory and Excitatory Signaling Complexes in Schizophrenia
Source: Neuron. 2015 Jun 3;86(5):1203–14. doi: 10.1016/j.neuron.2015.04.022 (PMC4460187; doi:10.1016/j.neuron.2015.04.022)
Supplement: Document S2. Article plus Supplemental Information [file mmc11.pdf]

Article

Neuron

Novel Findings from CNVs Implicate Inhibitory and Excitatory Signaling Complexes in Schizophrenia

Highlights

- First genetic evidence for disruption of GABAergic signaling in schizophrenia
- No evidence for CNV disruption of biological processes beyond the CNS
- Support for involvement of NMDAR and ARC complexes in schizophrenia
- Additional, independent evidence for disruption of glutamatergic signaling

Authors

Andrew J. Pocklington, Elliott Rees, ..., Michael C. O'Donovan, Michael J. Owen

Correspondence

pocklingtonaj@cardiff.ac.uk (A.J.P.), odonovanmc@cardiff.ac.uk (M.C.O.)

In Brief

Pocklington et al. show for the first time that CNVs from individuals with schizophrenia are enriched for genes involved in GABAergic neurotransmission. Previous findings of CNV enrichment among genes involved in glutamatergic signaling are independently replicated and greatly extended.

CrossMark

Pocklington et al., 2015, Neuron *86*, 1203–1214
June 3, 2015 ©2015 The Authors
http://dx.doi.org/10.1016/j.neuron.2015.04.022

CellPress

CellPress

Novel Findings from CNVs Implicate Inhibitory and Excitatory Signaling Complexes in Schizophrenia

Andrew J. Pocklington,[1,5,*] Elliott Rees,[1] James T.R. Walters,[1] Jun Han,[1] David H. Kavanagh,[1,4] Kimberly D. Chambert,[2] Peter Holmans,[1] Jennifer L. Moran,[2] Steven A. McCarroll,[2,3] George Kirov,[1] Michael C. O'Donovan,[1,5,*] and Michael J. Owen[1,5]
[1]MRC Centre for Neuropsychiatric Genetics and Genomics, Institute of Psychological Medicine and Clinical Neurosciences, Cardiff University, Cardiff CF14 4XN, UK
[2]Stanley Center for Psychiatric Research, The Broad Institute of MIT and Harvard, 75 Ames Street, Cambridge, MA 02142, USA
[3]Department of Genetics, Harvard Medical School, 77 Avenue Louis Pasteur, Boston, MA 02115, USA
[4]Current address: Division of Psychiatric Genomics in the Department of Psychiatry, Icahn School of Medicine at Mount Sinai, New York, NY 10029, USA
[5]Co-senior author
*Correspondence: pocklingtonaj@cardiff.ac.uk (A.J.P.), odonovanmc@cardiff.ac.uk (M.C.O.)
http://dx.doi.org/10.1016/j.neuron.2015.04.022
This is an open access article under the CC BY license (http://creativecommons.org/licenses/by/4.0/).

SUMMARY

We sought to obtain novel insights into schizophrenia pathogenesis by exploiting the association between the disorder and chromosomal copy number (CNV) burden. We combined data from 5,745 cases and 10,675 controls with other published datasets containing genome-wide CNV data. In this much-enlarged sample of 11,355 cases and 16,416 controls, we show for the first time that case CNVs are enriched for genes involved in GABAergic neurotransmission. Consistent with non-genetic reports of GABAergic deficits in schizophrenia, our findings now show disrupted GABAergic signaling is of direct causal relevance, rather than a secondary effect or due to confounding. Additionally, we independently replicate and greatly extend previous findings of CNV enrichment among genes involved in glutamatergic signaling. Given the strong functional links between the major inhibitory GABAergic and excitatory glutamatergic systems, our findings converge on a broad, coherent set of pathogenic processes, providing firm foundations for studies aimed at dissecting disease mechanisms.

INTRODUCTION

Schizophrenia is a highly heritable disorder (Cardno and Gottesman, 2000), the genetic architecture of which includes a large number of alleles spanning the full spectrum of frequencies (Sullivan et al., 2012). It has been estimated that the additive effects of common variation, as indexed by alleles represented on the platforms used in genome-wide association studies (GWASs), contribute around a quarter to a third of the total population variance in schizophrenia liability. However, the 108 genome-wide-associated common variant loci reported in the largest GWAS study to date only explain a small fraction of this contribution (Schizophrenia Working Group of the Psychiatric Genomics Consortium, 2014). An increased burden of rare mutations has also been documented in schizophrenia, taking the form of both large CNVs (International Schizophrenia Consortium, 2008; Rees et al., 2014b; Walsh et al., 2008) and single-nucleotide variants (SNVs) (Purcell et al., 2014), which often occur as de novo mutations (Kirov et al., 2012; Malhotra et al., 2011; Xu et al., 2008). While several CNVs have been implicated in the disorder, no individual SNV has yet been robustly associated (Purcell et al., 2014). The CNVs (n = 11) strongly associated with schizophrenia in the largest systematic survey to date (Rees et al., 2014b) are in general large in both size (> 500 kb) and effect (ORs 2–60), the latter being in stark contrast with the small effects conferred by common alleles (typical OR < 1.1). Approximately 2.5% of patients and 0.9% of unaffected controls carry a CNV that is strongly supported as a risk factor for schizophrenia (Rees et al., 2014b). The pathogenic effects of these CNVs are not confined to schizophrenia; many increase risk for other disorders with a putative major neurodevelopmental component such as intellectual disability, autism spectrum disorder, and attention deficit hyperactivity disorder (Girirajan et al., 2012; Malhotra and Sebat, 2012; Williams et al., 2010).

A small number of single-gene CNVs have been associated with schizophrenia, but the only ones to be definitively implicated are deletions of *NRXN1* (Kirov et al., 2008; Rees et al., 2014b), which encodes the presynaptic cell adhesion protein neurexin 1. All other robustly associated CNVs span multiple genes making it difficult to infer the biological mechanism(s) through which they contribute to disease. Nevertheless, early pathway or gene set analyses of schizophrenia case-control CNV datasets indicated that case CNVs were enriched for synaptic and neurodevelopmental genes (Glessner et al., 2010; Walsh et al., 2008).

It has been noted that these initial approaches to pathway analysis did not completely control for confounds such as CNV

CrossMark

size (Raychaudhuri et al., 2010). However, a study of parent-proband trios in which these factors were taken into account found that de novo CNVs in people with schizophrenia were enriched for synaptic proteins (Kirov et al., 2012). Moreover, this was largely the result of enrichment for genes encoding members of N-methyl-D-aspartate receptor (NMDAR) (Husi and Grant, 2001; Husi et al., 2000; Pocklington et al., 2006) and neuronal activity-regulated cytoskeleton-associated (ARC) protein complexes, both of which are known to be important for synaptic plasticity and cognitive function in rodents. When these same sets were additionally examined in large case-control datasets, case CNVs were found to contain an excess of genes from NMDAR, but not ARC, complexes (Kirov et al., 2012; Szatkiewicz et al., 2014). Exome sequencing studies have subsequently supported a role for both NMDAR and ARC complexes in disease (Fromer et al., 2014; Purcell et al., 2014). The same exome sequencing studies also found evidence of enrichment for rare disruptive and de novo point mutations among targets of fragile X mental retardation protein (FMRP) (Darnell et al., 2011), a finding that has also been reported for CNVs in a large schizophrenia case-control study (Szatkiewicz et al., 2014).

Here we present a detailed functional analysis of the largest schizophrenia CNV dataset for which full autosomal CNV data have been examined to date. The study is based on 11,355 cases and 16,416 controls from three separate studies: the International Schizophrenia Consortium (ISC), the Molecular Genetics of Schizophrenia (MGS), and a UK study of individuals diagnosed with schizophrenia and taking the anti-psychotic clozapine (CLOZUK) (International Schizophrenia Consortium, 2008; Levinson et al., 2011; Rees et al., 2014b). The ISC and MGS datasets were utilized in Kirov et al. (2012) to investigate CNV enrichment for ARC and NMDAR gene sets, while no CNV gene set analyses have yet been performed in CLOZUK. Starting from the hypothesis that schizophrenia reflects perturbation of brain function and development, our primary analysis focuses on a circumscribed set of annotations that are related to CNS function and development and are based on proteomic, RNA sequencing, and functional genetic data. In order to evaluate to what extent the pathogenic effects of CNVs primarily reflect disruption of brain function, as a secondary analysis we searched more widely for additional gene set enrichments using a more comprehensive range of annotations available from large, freely accessible databases.

RESULTS

We identified 134 gene sets relevant to various aspects of nervous system function and development, covering subcellular neuronal function, cellular physiology, cell morphology, brain region and fiber tract morphology, behavior, and brain development (Table S1). Gene sets were derived from functional studies of single genes recorded in the MGI Mammalian Phenotype (MP) database (Blake et al., 2014) with the exception of subcellular neuronal terms, which comprised a mixture of CNS-related gene sets taken from previous studies of schizophrenia (Fromer et al., 2014; Kirov et al., 2012; Purcell et al., 2014) as well as sets that were curated from the proteomic literature (see Table S1 for full list of references). To constrain multiple testing, we utilized a subset of the terms available from MGI (Table S1), which represented CNS annotations postulated to be of most likely relevance to schizophrenia, while at the same time retaining broad functional coverage to allow for the emergence of novel pathophysiological clues. Directional terms such as "decreased" or "enhanced" were avoided in favor of broader categories denoted by "abnormal," "impaired," etc.

CNV Enrichment in Gene Sets with Strong Prior Evidence for Involvement in Schizophrenia

Consistent with recent approaches (Szatkiewicz et al., 2014), our analyses are based on large, rare CNVs (> 100 kb, frequency < 1%), as these are both the most robustly called and most enriched in people with schizophrenia. Gene set enrichment analysis was performed using a logistic regression model (Kirov et al., 2012) with covariates included to control for the size and total number of genes overlapping each CNV and for the source of the data (study and genotyping array used). As we were only interested in gene sets that were enriched for CNVs in cases, we used one-tailed tests.

Of the 134 CNS-related gene sets, we first evaluated those for which there existed prior, replicated evidence of enrichment for rare mutations in schizophrenia in at least three independent studies: the NMDAR protein network, ARC protein complex, and mRNA targets of FMRP. Here, as in all subsequent analyses, we first tested for enrichment in the combined set of CNVs and then deletions and duplications separately. The results of these analyses were Bonferroni corrected for the nine tests performed. NMDAR network genes were highly enriched in CNVs overall ($P_{corrected} = 3.82 \times 10^{-8}$), the signal primarily coming from duplications ($P_{corrected} = 2.26 \times 10^{-8}$). The ARC gene set was not enriched for CNVs overall, but was enriched in the secondary test of deletions ($P_{corrected} = 0.0031$), while FMRP targets displayed a modest trend toward enrichment in deletions ($P_{corrected} = 0.076$).

Both ISC and MGS samples were utilized in our previous study (Kirov et al., 2012) to investigate CNV enrichment for ARC and NMDAR. We therefore asked whether we would have found the above NMDAR and ARC association signals if we had performed our analysis in CLOZUK only. Restricting to CLOZUK samples, case CNVs were still enriched for both NMDAR network ($P_{corrected} = 1.5 \times 10^{-6}$ combined, $P_{corrected} = 7.8 \times 10^{-7}$ duplications) and ARC ($P_{corrected} = 0.0077$ deletions) gene sets. Thus, our analysis provides fully independent evidence for the NMDAR network and ARC complexes.

Large CNVs Disrupt an Excess of CNS Gene Sets in Schizophrenia

We next investigated CNV enrichment for the full list of 134 CNS-related gene sets. To evaluate whether there was evidence for a general enrichment of CNS gene sets in our data, we tested whether the numbers of sets surpassing defined enrichment p value thresholds ($P_{uncorrected} < 0.01, 0.001$) were greater than expected. To do this, gene set enrichments for the full set of 134 CNS-related terms were compared to those generated from permuted data in which CNVs were randomly re-assigned among individuals, with the constraint that assignments were restricted to individuals from the same study so we could continue to allow for chip and study effects. In our analysis of

Table 1. Enrichment of CNS Gene Sets for Association Signal

			Significance Threshold							
			0.01				0.001			
	N_{case}	N_{ctrl}	N_{exp}	N_{obs}	p	P_{adj}	N_{exp}	N_{obs}	p	P_{adj}
All	8,139	10,469	1.3	23	< 0.001	< 0.006	0.2	13	< 0.001	< 0.006
Deletion	3,164	4,234	1.4	38	< 0.001	< 0.006	0.2	25	< 0.001	< 0.006
Duplication	4,975	6,235	1.4	14	0.004	0.024	0.2	10	0.001	0.006
All (minus known loci)	7,649	10,028	1.3	10	0.015	0.03	0.1	4	0.005	0.01
Deletion (minus known loci)	2,963	4,140	1.4	11	0.008	0.048	0.1	2	0.024	0.14
Duplication (minus known loci)	4,856	6,165	1.4	6	0.038	0.23	0.1	3	0.006	0.036

The number of CNS gene sets with association p value surpassing a pre-defined threshold ($p < 0.01$ or 0.001) was compared to that seen in permuted data (1,000 permutations of CNV case-control status). Columns list the number of case and control CNVs contributing to each analysis (N_{case} and N_{ctrl}, respectively); the average number of gene sets with p value surpassing a given threshold in the permuted data, N_{exp}; the actual number of gene sets surpassing the same threshold in the unpermuted data, N_{obs}; the empirical probability of finding N_{obs} or more gene sets surpassing the p value threshold in the permuted data, p; and the Bonferroni-corrected probability, P_{adj}. Results are given for the combined analysis of all CNVs and for the analysis of deletions and duplications separately; these are presented first for the full dataset and then for the subset of CNVs that do not overlap well-supported schizophrenia loci.

deletions and duplications combined, more sets were enriched for CNVs in schizophrenia than expected under the null at all enrichment p value thresholds. This was also true when deletions and duplications were considered separately (Table 1).

To evaluate the significance of the tests of individual gene sets in a manner that allows easy comparison with our later analyses of the much larger annotation datasets (where permutation tests were computationally prohibitive), we adjusted gene set p values for multiple testing using Bonferroni correction for the 402 CNS gene set tests (134 sets × 3 analyses) performed (Table S1). Recognizing that this is over-conservative due to annotation overlap, in Table 2 we additionally list all gene sets with an uncorrected $p < 0.001$ under the combined test of all CNVs. As can be seen from Table 1, given the large excess in the observed number of associated sets compared with expectation (minimum $N_{obs}/N_{exp} = 50$), most gene sets surpassing this threshold are likely to be true positives, even if they do not survive correction for multiple testing here. Functional processes captured by the six terms with a Bonferroni-corrected p value < 0.05 centered upon behavioral and physiological correlates of learning and related neuronal complexes.

For the combined analysis of all CNVs, after the NMDAR complex ($P_{corrected} = 1.71 \times 10^{-6}$) the next most highly associated term was the $GABA_A$ receptor complex ($P_{corrected} = 0.0012$). Conditional analyses revealed these two signals to be essentially independent (see conditional analysis of GO and MGI below; see also Supplemental Experimental Procedures). Thus not only do we confirm, as noted above, the involvement of proteins involved in plasticity of the major excitatory system of the CNS, we also provide the first strong genetic evidence for an etiological role in the disorder for proteins affiliated with the major inhibitory system in the CNS, namely $GABA_A$ receptor complexes (Heller et al., 2012).

Deletions and Duplications Independently Enriched in CNS Gene Sets

Tables 3 and 4 list all gene sets with $P_{uncorrected} < 0.001$ for enrichment within deletions and duplications, respectively. After Bonferroni correction for the 402 CNS gene set tests, there were 14 terms with p value < 0.05 for enrichment in case deletions and 7 terms for case duplications. Enrichment for duplications was largely confined to behavioral and subcellular neuronal gene sets; terms associated via deletions extended over behavior, cellular physiology, subcellular complexes, and development. Deletions were most highly enriched for components of PSD-95 protein complexes (Fernández et al., 2009). PSD-95, a major postsynaptic scaffolding protein at glutamatergic synapses, interacts with a wide range of channels and receptors including NMDARs. It is notable that although the NMDAR and PSD-95 complexes are functionally related and have overlapping membership, the observations of strong (ORs > 3) and highly significant enrichments (both $P_{corrected} < 10^{-7}$) for these sets relate to duplications and deletions, respectively. These findings are therefore based on sets of completely independent CNVs and, as such, provide extremely robust support for an etiological role for the disruption of glutamatergic signaling in schizophrenia.

Disruption of CNS Gene Sets Extends beyond Known Schizophrenia Loci

Current data provide strong support for 11 CNV loci in schizophrenia: 6 deletions and 5 duplications (Rees et al., 2014b) (Table S2). Removing CNVs overlapping these known loci, we re-calculated CNS gene set enrichment. Deletion, duplication, and combined analyses all retained an excess of associated terms (Table 1); of the 14 gene sets enriched for deletions, 5 remained nominally associated ($P_{uncorrected} < 0.05$) when known loci were removed, as did 5 of the 7 terms enriched for duplications (Table S3).

Individual Genes within Associated Gene Sets

To identify genes contributing most to gene set enrichment we calculated single gene association p values. This was done in the same manner as our gene set enrichment analyses, but with each "set" restricted to a single gene. For each CNS term with a Bonferroni-corrected p value < 0.05 we then extracted

Table 2. Enriched CNS Gene Sets, Combined Analysis

		Combined			Deletion		Duplication	
	N_{gene}	p	P_{adj}	OR (95% CI)	p	P_{adj}	p	P_{adj}
NMDAR network	59	4.3×10^{-9}	1.7×10^{-6}	2.47 (1.8–3.44)	0.045	1	2.5×10^{-9}	1.0×10^{-6}
$GABA_A$	15	3.0×10^{-6}	0.0012	2.51 (1.65–3.97)	0.00068	0.27	5.4×10^{-5}	0.022
Abnormal associative learning	193	1.6×10^{-5}	0.0066	1.38 (1.19–1.61)	1.0	1	1.6×10^{-10}	6.2×10^{-8}
Abnormal long-term potentiation	145	2.0×10^{-5}	0.0081	1.49 (1.24–1.8)	0.58	1	1.1×10^{-6}	0.00044
Abnormal behavior	1,973	5.1×10^{-5}	0.020	1.12 (1.06–1.19)	3.0×10^{-6}	0.0012	0.05	1
Abnormal CNS synaptic transmission	371	5.5×10^{-5}	0.022	1.22 (1.11–1.35)	5.1×10^{-6}	0.002	0.12	1
Thin cerebral cortex	45	0.00018	0.071	1.91 (1.32–2.8)	0.12	1	0.0006	0.24
Abnormal consumption behavior	442	0.00019	0.077	1.24 (1.09–1.41)	0.059	1	0.0005	0.2
Abnormal cued conditioning behavior	68	0.00027	0.11	1.69 (1.24–2.35)	0.55	1	1.4×10^{-5}	0.0055
Abnormal synaptic transmission	437	0.00027	0.11	1.18 (1.08–1.29)	1.1×10^{-5}	0.0044	0.21	1
Abnormal learning/memory/conditioning	424	0.00031	0.12	1.18 (1.08–1.29)	7.3×10^{-5}	0.029	0.089	1
PSD-95 (core)	58	0.00048	0.19	1.71 (1.28–2.28)	4.3×10^{-11}	1.7×10^{-8}	0.97	1
Abnormal contextual conditioning behavior	89	0.00061	0.24	1.53 (1.18–1.99)	0.52	1	0.00011	0.045

CNS gene sets with $P_{uncorrected} < 0.001$ in the combined analysis of deletions and duplications are listed along with the number of genes in each set, N_{gene}; uncorrected (p) and Bonferroni-corrected (P_{adj}) p values for enrichment in case CNVs; estimated odds ratios (OR); and p values for enrichment in case deletions and duplications when analyzed separately. Note that while the NMDAR network was analyzed prior to other terms in this table, here it is corrected for the same number of tests as other terms for ease of comparison. See also Tables S1 and S3.

all genes with an uncorrected single gene p value < 0.05 (Tables S4, S5, and S6). To obtain significance at the level of an individual gene, there must be multiple observations of CNVs at the same region. It is therefore unsurprising that established recurrent CNV risk loci account for many such findings. Moreover, as recurrent CNVs are large, these frequently overlap multiple genes and contribute to multiple sets (e.g., del22q11; Table S5). It should be noted that some CNVs also hit multiple genes within a single set, but as each CNV only contributes once to the regression model (see Experimental Procedures), co-localization of set members does not inflate the significance of the set-based enrichment. A number of nominally associated genes lying outside established loci are well known to be important for neuronal signaling. These include the glutamate transporter *SLC1A1*, a recently reported candidate CNV locus for schizophrenia (Myles-Worsley et al., 2013; Rees et al., 2014a); GABAergic (*GABRD* also reported in Rees et al., 2014a) and nicotinic receptors (*CHRNA4*); synaptic scaffolding proteins *DLG2*, *DLGAP1*, and *SHANK2*; and key elements of the presynaptic vesicle release machinery *PCLO* and *NSF*.

Some of the nominally associated genes have been linked to Mendelian disorders with neurological symptoms, including Walker-Warburg syndrome, a congenital muscular dystrophy with brain and eye abnormalities (*ISPD* OMIM: 614643, *POMK* OMIM: 615249); nocturnal frontal lobe epilepsy type 1 (*CHRNA4* OMIM: 600513); generalized epilepsy (*GABRD* OMIM: 613060); spastic paraplegia 51, an autosomal recessive developmental disorder with severe intellectual disability (*AP4E1* OMIM: 613744); and Batten disease, an autosomal recessive neurodegenerative condition (*CLN3* OMIM: 204200).

No Evidence for Gene Set Enrichment beyond CNS

We next determined whether any other gene sets had evidence for enrichment in case CNVs that was independent of the association signal captured by our primary CNS-related terms. For this we drew upon both the MGI MP database (Blake et al., 2014), from which we had derived most of our CNS-related gene sets, and the widely used Gene Ontology (GO) terms (Ashburner et al., 2000). As the MP database contains an extensive range of physiological, behavioral, and morphological phenotypes, but little of the low-level molecular function annotation present in GO, these two classifications are to an extent nonredundant and complementary.

We first identified a "minimal set" of terms capturing most of the enrichment signal arising from CNS-related gene sets. Taking the CNS terms surviving Bonferroni correction, we added the most significant term as a covariate to the regression model and recalculated gene set enrichment for each of the remaining terms. The term with the most significant residual enrichment was then added to the model, and the process repeated until there was no residual association ($P_{uncorrected} < 0.05$) in the remaining CNS annotations. Three terms were required to capture CNS gene set enrichment in the combined analysis of duplications and deletions: NMDAR network, $GABA_A$ receptor complex, and abnormal behavior (see Supplemental Experimental Procedures). This indicates that there are independent enrichment signals in the NMDAR network and $GABA_A$ receptor complex gene sets, as reported above. Conditioning on the above three CNS terms, no other GO or MP term survived Bonferroni correction (Tables S7 and S8).

CNS gene set enrichment for deletions was captured by three terms: PSD-95 complex, abnormal fear/anxiety-related behavior, and abnormal neural plate morphology (see Supplemental Experimental Procedures). Conditioning on these terms, no MP or GO term survived Bonferroni correction (Tables S7 and S8). Three terms captured CNS enrichment in duplications: associative learning, NMDAR network, and $GABA_A$ receptor complexes (see Supplemental Experimental Procedures).

Table 3. Enriched CNS Gene Sets, Deletions

	N_{gene}	p	P_{adj}	OR (95% CI)
PSD-95 (core)	58	4.3×10^{-11}	1.7×10^{-8}	4.62 (2.85–7.8)
Abnormal neural plate morphology	23	2.1×10^{-7}	8.4×10^{-5}	
Abnormal prepulse inhibition	74	3.3×10^{-7}	0.00013	1.94 (1.46–2.76)
Abnormal behavior	1,973	3.0×10^{-6}	0.0012	1.35 (1.2–1.54)
Abnormal fear/anxiety-related behavior	216	3.2×10^{-6}	0.0013	1.74 (1.38–2.23)
Abnormal CNS synaptic transmission	371	5.1×10^{-6}	0.002	1.56 (1.29–1.92)
Abnormal spatial working memory	38	5.6×10^{-6}	0.0022	4.94 (2.33–14.56)
Abnormal synaptic transmission	437	1.1×10^{-5}	0.0044	1.46 (1.23–1.74)
Abnormal emotion/affect behavior	369	1.1×10^{-5}	0.0044	1.45 (1.23–1.75)
Abnormal neuron differentiation	206	2.8×10^{-5}	0.011	2.51 (1.67–3.87)
Abnormal spatial learning	156	4.8×10^{-5}	0.019	1.66 (1.3–2.12)
Abnormal social/conspecific interaction	243	4.8×10^{-5}	0.019	1.56 (1.26–1.97)
Abnormal learning/memory/conditioning	424	7.3×10^{-5}	0.029	1.44 (1.21–1.73)
Abnormal miniature excitatory postsynaptic currents	62	0.0001	0.041	2.74 (1.57–4.95)
Cav2_channels	202	0.00017	0.068	1.85 (1.33–2.59)
Abnormal excitatory postsynaptic currents	69	0.00025	0.10	1.95 (1.31–2.93)
Abnormal axon extension	46	0.00027	0.11	5.68 (2.21–17.65)
Abnormal depression-related behavior	76	0.00033	0.13	3.69 (1.75–8.54)
ARC	25	0.00034	0.14	1.7 (1.24–2.33)
Abnormal excitatory postsynaptic potential	59	0.00067	0.27	4.2 (1.64–12.87)
$GABA_A$	15	0.00068	0.27	2.43 (1.36–4.49)
Abnormal nervous system development	801	0.00073	0.29	1.43 (1.17–1.75)
Abnormal aggression-related behavior	63	0.00075	0.30	3.33 (1.64–7.24)
Abnormal response to novelty	152	0.00079	0.32	1.48 (1.18–1.87)
Abnormal sensory capabilities/reflexes/nociception	590	0.0008	0.32	1.39 (1.13–1.7)

CNS gene sets with $P_{uncorrected} < 0.001$ in the analysis of deletions are listed along with number of genes in each set, N_{gene}; uncorrected (p) and Bonferroni-corrected (P_{adj}) p values for enrichment in case CNVs; and estimated odds ratios (OR). See also Tables S1 and S3.

Once again there was no evidence of additional gene set enrichment in either MP or GO annotations following Bonferroni correction (Tables S7 and S8).

Pathogenicity of Large CNVs Is Related to the Number of CNS Genes Hit

Case CNVs > 100 kb were both larger ($P_{del} = 6.8 \times 10^{-14}$, $P_{dup} = 0.37$) and overlap ("hit") a greater number of genes ($P_{del} = 1.3 \times 10^{-16}$, $P_{dup} = 3.4 \times 10^{-5}$) than those found in controls. However, even after conditioning on CNV size, the number of genes hit was strongly and independently associated ($P_{del} = 1.0 \times 10^{-5}$, $P_{dup} = 2.9 \times 10^{-5}$), whereas after conditioning on number of genes, the effect of size was much weaker and was restricted to deletions ($P_{del} = 0.0073$, $P_{dup} = 0.49$). The number of genes hit is therefore a better predictor of case-control status than CNV size.

We next investigated whether the relationship between number of genes hit and case-control status could be entirely attributed to genes within the disease-associated CNS annotations. To test this, we combined all the CNS annotations that had a Bonferroni-corrected p value < 0.05 to create a single associated CNS set (CNS_{SZ}). We did this separately for deletions and duplications. The number of CNS_{SZ} genes hit by a CNV was a highly significant predictor of case-control status for both deletions and duplications ($P_{del} = 1.1 \times 10^{-21}$, $P_{dup} = 1.7 \times 10^{-12}$). Each was at least five orders of magnitude more significant than the corresponding analyses based on total number of genes hit (see previous paragraph). As expected this CNS_{SZ} term remained highly significant when conditioned on total number of genes hit in any category ($P_{del} = 7.7 \times 10^{-7}$, $P_{dup} = 1.1 \times 10^{-9}$), but the converse was not the case; conditioning on CNS_{SZ} there was little evidence of any remaining effect of the total number of genes hit by each CNV ($P_{del} = 0.21$, $P_{dup} = 0.053$).

CNV Association Identifies Gene Sets Enriched for Rare, De Novo NS Mutations

Finally, we investigated whether associated CNS gene sets were also enriched in de novo non-synonymous (NS) mutations (Fromer et al., 2014). To constrain both the number and size of the gene sets tested, we collapsed the "minimal set" of terms capturing most of the CNS enrichment signal (see analysis of GO and MGI above) into a single gene set for each of our analyses (combined, deletion only, duplication only). Gene sets capturing the CNS enrichments for deletions and duplications were associated with de novo NS mutations observed in individuals with schizophrenia. This was not entirely due to ARC and NMDAR network genes (which we have previously described

Table 4. Enriched CNS Gene Sets, Duplications

	N_{gene}	p	P_{adj}	OR (95% CI)
Abnormal associative learning	193	1.6×10^{-10}	6.2×10^{-8}	1.73 (1.46–2.08)
NMDAR network	59	2.5×10^{-9}	1.0×10^{-6}	3.09 (2.09–4.67)
Abnormal long-term potentiation	145	1.1×10^{-6}	0.00044	1.65 (1.34–2.04)
Abnormal avoidance learning behavior	56	1.6×10^{-6}	0.00066	1.89 (1.45–2.47)
Abnormal cued conditioning behavior	68	1.4×10^{-5}	0.0055	2.02 (1.41–3)
$GABA_A$	15	5.4×10^{-5}	0.022	2.8 (1.56–5.67)
Abnormal contextual conditioning behavior	89	0.00011	0.045	1.68 (1.28–2.23)
Abnormal consumption behavior	442	0.00050	0.20	1.27 (1.1–1.46)
Abnormal temporal memory	108	0.00052	0.21	1.56 (1.2–2.04)
Thin cerebral cortex	45	0.00060	0.24	1.94 (1.3–2.92)

CNS gene sets with $P_{uncorrected} < 0.001$ in the analysis of duplications are listed along with number of genes in each set, N_{gene}; uncorrected (p) and Bonferroni-corrected (P_{adj}) p values for enrichment in case CNVs; and estimated odds ratios (OR). Note that while the NMDAR network was analyzed prior to other terms in this table, here it is corrected for the same number of tests as other terms for ease of comparison. See also Tables S1 and S3.

to be enriched for de novo NS mutations; Fromer et al., 2014) (Table 5). No enrichment was found when the analysis was repeated in a corresponding set of mutations from unaffected individuals (Table 5). When all 21 CNS terms with $P_{corrected} < 0.05$ (Tables 2–4) were tested individually, over half were nominally enriched for de novo NS mutations (cf. none for mutations from controls). While none survive correction for multiple testing (Table S9), this has to be interpreted in the context of the very weak enrichment in schizophrenia for de novo NS mutations, and therefore low power to robustly detect gene set enrichment. These findings independently support the broader relevance of the gene sets we have identified in the present study, but larger studies of de novo mutations will be required for finer-scale dissection.

DISCUSSION

We have performed a detailed, functionally informed analysis of large, rare CNVs from 11,355 schizophrenia cases and 16,416 controls. The results provide strong, novel evidence implicating disruption of inhibitory GABAergic modulation of neuronal signaling in schizophrenia and robustly confirm, and extend, the genetic evidence implicating disruption of excitatory glutamatergic signaling (Figure 1). It is clear, however, that these neuronal complexes do not entirely account for the enrichment of CNVs in cases given the independent enrichments seen in behavioral and neurodevelopmental gene sets. This suggests that subcellular processes beyond those currently ascribed to GABAergic and glutamatergic complexes remain to be identified.

We found no evidence that the pathogenic effects of CNVs reflect biological processes other than those directly relevant to brain function. This conclusion follows from the absence of additional gene set enrichments after conditioning on the CNS sets, and is further supported by the observation that the number of genes hit by a CNV in the disease-associated CNS pathways was a better predictor of whether a CNV occurred in a case or a control than total number of genes hit. This contrasts with recent findings based on common polymorphisms, where independent enrichments were found in enhancer elements that were active in both CNS and immune tissues (Schizophrenia Working Group of the Psychiatric Genomics Consortium, 2014), although a more recent analysis by that group suggests that most, if not all, of the signal is captured by the CNS enhancers (http://biorxiv.org/content/early/2015/01/23/014241).

While 11 CNV loci have been strongly associated with schizophrenia to date (Rees et al., 2014b), our results indicate that more associated CNVs remain to be identified. The association between number of genes hit and pathogenicity suggests that, in many instances, looking for a single gene explanation for CNV pathogenicity may not be fruitful. Instead it indicates that pathogenicity depends upon the total burden of relevant genes hit by a CNV, or that different single genes are implicated in different individuals depending upon their genetic and environmental context—the larger the CNV, the greater the probability that a critical pathway or process will be sufficiently impaired. It should be noted that the presence of multiple hits in the same CNV does not artificially inflate the significance of our enrichment tests, as each CNV only contributes once to the analysis.

There were strong, independent associations in postsynaptic complexes derived from glutamatergic synapses: NMDAR complex (Husi and Grant, 2001; Husi et al., 2000; Pocklington et al., 2006) genes were enriched in case duplications, while PSD-95 (Fernández et al., 2009) and to a lesser extent ARC complexes (Kirov et al., 2012) were enriched in deletions. When these findings are combined with existing evidence from de novo CNVs (Kirov et al., 2012), from rare SNVs and indels (Fromer et al., 2014; Purcell et al., 2014), and more recently from GWASs and common alleles (Schizophrenia Working Group of the Psychiatric Genomics Consortium, 2014), the relevance of altered glutamatergic signaling to schizophrenia etiology and pathophysiology seems to be beyond any reasonable doubt.

While models of schizophrenia based upon NMDAR hypofunction have a long history (Olney and Farber, 1995), the genetic data now indicate that the glutamatergic contribution to schizophrenia encompasses a much wider range of cellular processes converging upon synaptic information processing and plasticity (Figure 1). This is clearly inconsistent with hypotheses in which deficits in glutamatergic signaling primarily reflect disruption via neuromodulatory pathways (Stephan et al., 2009). Genetic evidence for disruption of neuromodulators is so far restricted to dopamine, with a genome-wide significant GWAS signal localized to *DRD2* (Schizophrenia Working Group of the Psychiatric Genomics Consortium, 2014). Both serotonergic 5-$HT2_C$ and nicotinic α_7 receptor complexes were tested here, and neither was found to be strongly associated (Table S1).

Table 5. "Minimal" CNS Gene Sets, Enrichment for NS De Novo Rare Variants

	N_{gene}	De Novo SNV	N Mutation Observed	N Mutation Expected	p	P_{adj}	Minus ARC/NMDAR N_{gene}	Minus ARC/NMDAR p
Combined	1,991	schizophrenia	110	96.63	0.084	0.24	1,930	0.25
Deletion	287		27	13.96	0.0014	0.0042	255	0.011
Duplication	249		24	12.04	0.0015	0.0045	191	0.026
Combined	1,991	control	64	60.45	0.33	1	1,930	0.28
Deletion	287		10	8.81	0.39	1	255	0.24
Duplication	249		6	7.53	0.76	1	191	0.68

Gene sets capturing CNS enrichment in combined, deletion, and duplication analyses were tested for enrichment with rare, non-synonymous de novo mutations from individuals with schizophrenia. Listed are number of genes in each gene set (N_{gene}); number of variants found within these genes (Observed); number of variants expected (Expected); uncorrected and Bonferroni-corrected p values (p, P_{adj}), where correction is for the three gene sets tested; plus p values following removal of ARC and NMDAR genes (Minus ARC/NMDAR). Analysis was then repeated for NS de novo rare variants identified in unaffected controls (same correction procedure). See also Table S9.

We also find novel, independent evidence for disruption of $GABA_A$ receptor complexes (Heller et al., 2012) in schizophrenia. Deficits in GABAergic signaling have long been hypothesized to contribute to schizophrenia pathophysiology alongside perturbation of dopaminergic and glutamatergic systems (Carlsson, 1988; Lewis et al., 2012; Olney and Farber, 1995; Roberts, 1972). Evidence supporting a direct involvement of GABA has as yet not been compelling, being drawn from imaging studies and animal models of putative intermediate phenotypes, or post-mortem expression studies in small samples where reverse causality or confounding cannot be excluded (Inan et al., 2013). Here we find case CNVs to be enriched for components of $GABA_A$ receptor complexes (Tables 2–4), with conditional analyses revealing the $GABA_A$ association signal to be independent of that seen for NMDAR complex genes. Our results indicate that abnormalities in GABAergic signaling play a direct pathogenic role in schizophrenia and cannot be entirely attributed to secondary effects of NMDAR dysfunction (Laruelle et al., 2005; Lewis and Gonzalez-Burgos, 2006).

$GABA_A$ receptor complex enrichment was strongest among duplications, where the most highly associated genes were $\alpha5$, $\beta3$, and δ receptor subunits (Table S10). The genes encoding $\alpha5$ and $\beta3$ subunits are found within the Angelman/Prader-Willi locus, while the δ subunit has been mapped to the critical region for the 1p36 deletion syndrome: a relatively common CNV associated with a range of neurodevelopmental outcomes (Battaglia et al., 2008; Shapira et al., 1997; Windpassinger et al., 2002) that has recently been identified as a candidate locus for schizophrenia (Rees et al., 2014a). The remaining autosomal $GABA_A$ receptor genes largely cluster within two loci on chromosomes 4 and 5, neither of which displayed evidence of enrichment (Table S10). The $GABA_A$ enrichment signal for deletions was driven by *NRXN1*, which encodes for the presynaptic cell adhesion protein neurexin 1, common to both GABAergic and glutamatergic synapses.

Multiple $GABA_A$ receptor subtypes exist, each with a unique set of functional properties and a distinct spatiotemporal expression profile (reviewed by Fritschy and Panzanelli, 2014). In contrast to the $\beta3$ subunit, which is common to many receptor subtypes, $\alpha5$ and δ subunits occur in distinct, mainly extrasynaptic populations of receptors responsible for tonic inhibition. This indicates that the contribution of GABAergic signaling to schizophrenia may not be primarily synaptic, although the presence of CNVs in *NRXN1* and *GHPN*, encoding neurexin 1 and the synaptic GABA receptor scaffolding protein gephyrin (Table S10), suggests that perturbation of synaptic GABAergic signaling may also play a role.

Tonic inhibition in the hippocampus, a process to which both $\alpha5$- and δ-containing $GABA_A$ receptors contribute (Glykys et al., 2008), alters the induction of long-term potentiation (Martin et al., 2010). Moreover, prolonged activation of NMDARs has been shown to reduce cell surface expression of δ-containing receptors (Joshi and Kapur, 2013). Thus, there are potential functional connections between our findings of enrichment for CNVs in GABAergic and postsynaptic glutamatergic complexes in schizophrenia. At the behavioral level, perturbation of NMDAR and tonic GABA signaling both lead to alterations in associative learning (Bauer et al., 2002; Martin et al., 2010; Rodrigues et al., 2001). This hierarchy of functionally related processes, from subcellular complexes to behavioral learning via cellular signaling and plasticity, encapsulates the elements of gene set enrichment common to case duplications and deletions (Tables 2–4).

Our findings are consistent with a considerable body of nongenetic literature; it is nearly 40 years since the cognitive deficits seen in schizophrenia were first proposed to reflect dysfunctional associative learning (Miller, 1976), with later hypotheses suggesting perturbed synaptic plasticity as the source of this dysfunction (Friston, 1998). Our identification of independent genetic associations in glutamatergic and GABAergic complexes is particularly relevant to proposals that alteration in the ratio of excitatory to inhibitory transmission (E/I balance) underlies the behavioral deficits seen in schizophrenia (reviewed by Kehrer et al., 2008; see also Yizhar et al., 2011). Discussions typically focus upon the oscillatory properties of neuronal networks, fundamental to efficient information transfer and the coordination of neuronal assemblies (Buzsáki and Draguhn, 2004; Buzsáki and Watson, 2012). Deficits in gamma rhythms have been reported in schizophrenia (reviewed by Uhlhaas and Singer, 2010), and while multiple mechanisms contribute to the generation of these rhythms (Bartos et al., 2007), deficits have primarily been hypothesized to result from the altered firing of GABAergic interneurons (Lewis and Gonzalez-Burgos, 2006; Uhlhaas and

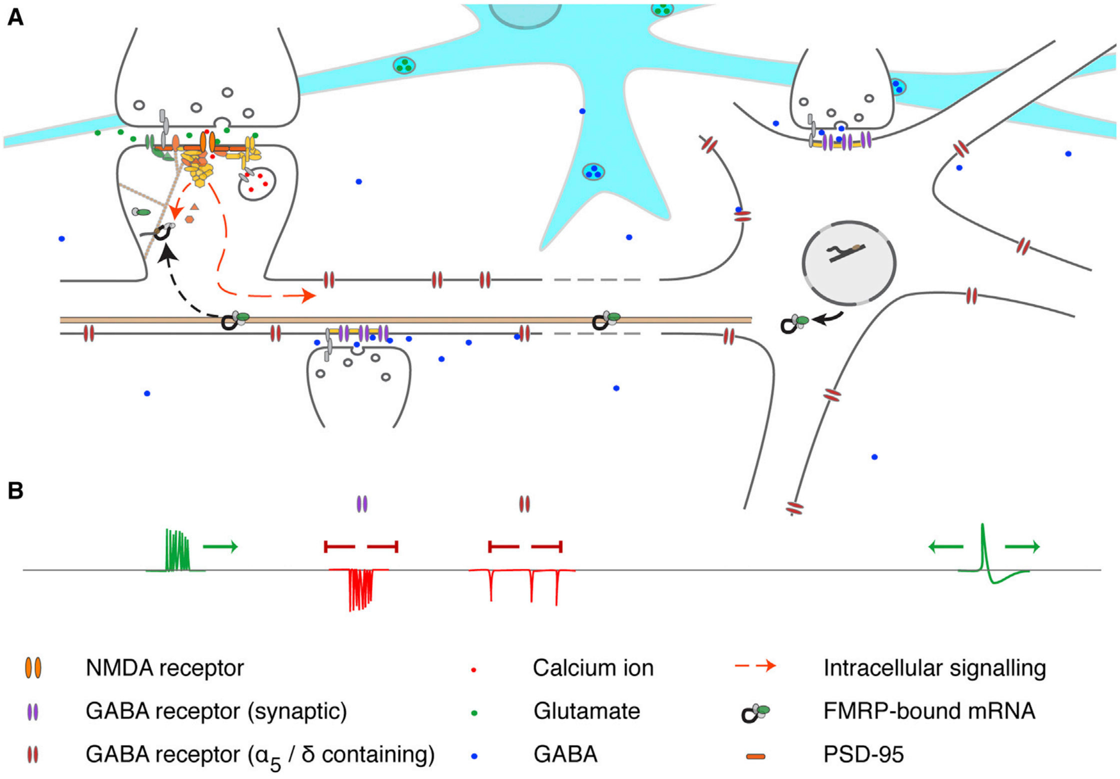

Figure 1. Functional Interactions between Neuronal Complexes Implicated in Schizophrenia

Supporting and extending previous studies (Fromer et al., 2014; Kirov et al., 2012; Purcell et al., 2014), our analyses indicate a contribution to schizophrenia from ARC, NMDAR network, PSD-95, and $GABA_A$ neuronal complexes. Although not strongly associated here, targets of the translational repressor FMRP have previously been found to be enriched in CNVs and rare de novo small mutations in individuals with schizophrenia (Fromer et al., 2014; Purcell et al., 2014; Szatkiewicz et al., 2014). This figure summarizes the relationship between these sets of molecules and their roles in synaptic signaling and plasticity.

(A) PSD-95 complexes are an important component of the postsynaptic scaffold at glutamatergic synapses, linking a wide range of channels and receptors including NMDARs (top left). Calcium influx via the NMDAR drives multiple downstream pathways (red arrows): local signaling regulates induction of synaptic potentiation, while activation of ARC transcription via signaling to the nucleus is required for the long-term maintenance of synaptic changes. Once transcribed, mRNAs encoding ARC and other synaptic proteins are inactivated via association with FMRP and transported to synaptodendritic sites of protein synthesis. Here, activity-dependent dissociation of FMRP releases transcripts from translational repression allowing protein synthesis and incorporation into active synapses.

(B) NMDAR activation requires both presynaptic glutamate release and strong post-synaptic depolarization, which may be induced by the back-propagation of action potentials. Influx of chloride ions via GABA receptors attenuates the dendritic transmission of excitation, inhibiting action potential generation and back-propagation. Phasic firing of synaptic GABA receptors plays a key role in establishing neural oscillations, required for the coordination of distributed functional networks. Tonic GABA receptors also modulate excitatory currents and oscillatory neuronal behavior, being responsive to local network activity via the overspill of GABA from synaptic receptors and its release/uptake by glia (blue cell in A). For simplicity all receptors are shown acting upon a single neuron; in reality, their interplay is distributed across multiple neuronal cell types, e.g., tonic GABA currents also modulating synaptic GABA release from interneurons.

Singer, 2010). Interestingly, the frequency of hippocampal gamma oscillations is sensitive to the balance between NMDAR-dependent excitation and GABA δ subunit-dependent tonic inhibition of interneurons (Mann and Mody, 2010), linking these hypotheses directly to our findings.

Although less discussed in relation to schizophrenia, E/I balance also plays a role in the development and maintenance of stable perceptual and motor representations (reviewed by Carcea and Froemke, 2013). During early post-natal development unbalanced excitatory input drives activity-dependent plasticity, shaping emerging networks of synaptic connections in response to the environment. As networks mature and inhibitory elements are progressively integrated, E/I inputs become correlated making internal representations resistant to further modification. In the adult brain, activation of neuromodulatory systems (including acetylcholine, dopamine, and serotonin) can alter E/I balance, allowing sensory circuits to again become sensitive to environmental input. Unlike the experience-dependent modifications that occur during development, plasticity in adult sensory cortex is generally transient (see Carcea and Froemke, 2013). Genetic associations potentially link the main elements of these processes to schizophrenia: E/I signaling and synaptic plasticity through our analyses, dopaminergic signaling via the *DRD2* GWAS locus noted above (Schizophrenia Working Group of the Psychiatric Genomics Consortium, 2014). Disruption of such processes may potentially play a role in the developmental trajectory of schizophrenia, or in the manifestation of transient perceptual alterations during psychotic episodes.

In conclusion, our analyses support and extend previous studies (Fromer et al., 2014; Kirov et al., 2012; Purcell et al., 2014) indicating a contribution to schizophrenia from complexes central to the induction (NMDAR) and maintenance (ARC) of synaptic plasticity and provide strong novel evidence for the involvement of inhibitory modulation (GABA) of synaptic signaling (Figure 1). Perturbation of these processes is likely to have a widespread impact on brain function, and only a subset of genetic lesions within these systems may be compatible with a schizophrenia phenotype. The identification of the mechanisms by which disruption of these processes by genetic mutation leads to psychopathology will doubtless require experimental studies in model systems of high construct validity. The strength of genetic evidence converging on a plausible and coherent set of biological processes provides firm foundations upon which such studies can now proceed.

EXPERIMENTAL PROCEDURES

Samples, Genotyping, and CNV Quality Control

Case and control CNVs were derived from three samples: CLOZUK, the ISC, and the MGS. A full description of these samples, the arrays they were genotyped on, and CNV calling procedures can be found in the Supplemental Experimental Procedures or in the original publications (International Schizophrenia Consortium, 2008; Levinson et al., 2011; Rees et al., 2014a). Briefly, CLOZUK samples were genotyped on several Illumina arrays. In order to limit any bias in detecting specific CNVs between the CLOZUK cases and controls, done on different arrays, for CNV calling we used only the 520,766 probes common to all these arrays. In the CLOZUK sample CNVs were called with PennCNV (Wang et al., 2007). All MGS samples were genotyped on Affymetrix 6.0 arrays, and approximately equal proportions of ISC cases and controls were genotyped on either Affymetrix 6.0 or Affymetrix 5.0 arrays (see Supplemental Experimental Procedures). In the MGS and ISC samples CNVs were detected using Birdsuite (Korn et al., 2008). Only samples with a European ancestry were retained for analysis. Rigorous quality control was performed to remove low-quality samples (full details presented in the Supplemental Experimental Procedures), resulting in 5,745 cases and 10,675 controls in CLOZUK, 2,214 cases and 2,556 controls in MGS, and 3,395 cases and 3,185 controls in ISC retained for analysis. Taking CNV calls from samples which passed quality control in each study, CNVs were joined if the distance separating them was less than 50% of their combined length. CNVs were excluded if they overlapped low copy repeats by more than 50% of their length, or had a probe density < 1 probe/20 kb. CNVs with a frequency > 1% or identified as false positives by an in silico median *Z* score outlier method were also removed (Kirov et al., 2012). *Z* score validation was not performed for the ISC study as we did not have access to the raw intensity data. Following QC, genes overlapping CNVs were identified using genomic locations for the appropriate build of the human genome: Build 35 of the human genome for ISC, Build 36 for MGS, and Build 37 for CLOZUK. Studies were then collated, and CNVs < 100 kb in size and/or covered by < 15 probes were removed prior to analysis. Differences in genotyping chip, CNV calling, and genome build between studies are controlled for in our enrichment analyses through the "chip" and "study" covariates; while between-study differences may reduce power to identify true positives, they do not increase the rate of false positives. See Supplemental Experimental Procedures for further details.

Gene Annotations

Proteomic studies used to derive subcellular terms are listed in Table S1. For terms analyzed in Kirov et al. (2012), the processed gene sets analyzed in that study were re-used here. Gene sets for all other subcellular terms were extracted from the relevant studies and mapped to human coding genes. GO annotations were taken from NCBI gene2go (ftp://ftp.ncbi.nih.gov/DATA), using *Homo sapiens* annotations only. MP ontology and gene annotations were downloaded from the Mouse Genome Informatics (MGI) online resource (http://www.informatics.jax.org). Genes were mapped to human using the file HOM_MouseHumanSequence.rpt, also downloaded from MGI. For further details see Supplemental Experimental Procedures.

Enrichment Test for Individual Gene Sets

For each gene set, the numbers of genes "hit" by case and control CNVs were compared; a gene was counted as being hit by a CNV if the CNV overlapped any part of its length. To overcome biases related to gene and CNV size, and to control for differences between studies and genotyping chips, the following logistic regression models were fitted to the combined set of CNVs:

(a) logit (pr(case)) = study + chip + CNV size + total number of genes hit
(b) logit (pr(case)) = study + chip + CNV size + total number of genes hit + number of genes hit in gene set

Comparing the change in deviance between models (a) and (b), a one-sided test for an excess of genes in the gene set being hit by case CNVs was performed. For further details and a full description of the approach taken for multiple testing correction (outlined in the main text), see Supplemental Experimental Procedures.

Permutation Test for General Enrichment of CNS Gene Sets

Case-control status was permuted 1,000 times, status being shuffled between CNVs from the same study and genotyping chip ("Affymetrix 5.0," "Affymetrix 6.0," or "Illumina"). Enrichment analyses were performed in each permuted dataset; the proportion of datasets in which the number of terms with $p < P_{thr}$ equaled or exceeded that of the true data being used as the empirical p value for an excess of associated terms at the threshold P_{thr}.

Enrichment beyond CNS-Related Terms

From CNS gene sets with $P_{corrected} < 0.05$, a subset was identified that captured the association signal in all other terms. GO and MGI terms were then analyzed using the enrichment test outlined above, but with this "minimal" set of terms added as covariates to the regression models (see main text and Supplemental Experimental Procedures).

Removing Signal from Known Loci

To investigate whether gene set enrichment was solely driven by CNVs at loci well supported by current data (Table S2), we removed all CNVs overlapping these loci and re-ran the enrichment analysis as above. See Supplemental Experimental Procedures for further details.

Single Gene Enrichment Analysis

This was performed in an identical manner to gene set enrichment analysis, but with each "gene set" here comprising a single gene. The term "number of genes hit in gene set" in model (b) thus becomes a binary variable.

CNV Size and Number of Genes Hit as Predictors of Case-Control Status

CNV size and number of genes hit (either total or CNS_{SZ}) were regressed against CNV case-control status under a logistic regression model. Covariates were included for study and genotyping chip (as in enrichment test, above). See Supplemental Experimental Procedures for further details.

De Novo Rare Variant Analysis

NS de novo variants found in individuals with schizophrenia were taken from Fromer et al. (2014), consisting of variants identified in four separate studies (Fromer et al., 2014; Girard et al., 2011; Gulsuner et al., 2013; Xu et al., 2012). These were analyzed for gene set enrichment using the dnenrich software (Fromer et al., 2014) (http://bitbucket.org/statgen/dnenrich). NS de novo variants found in unaffected individuals were also taken from Fromer et al. (2014) and analyzed in an identical fashion. These consisted of healthy controls and unaffected siblings collated from six separate studies (Gulsuner et al., 2013; Iossifov et al., 2012; O'Roak et al., 2012; Rauch et al., 2012; Sanders et al., 2012; Xu et al., 2012).

SUPPLEMENTAL INFORMATION

Supplemental Information includes ten tables and Supplemental Experimental Procedures and can be found with this article online at http://dx.doi.org/10.1016/j.neuron.2015.04.022.

AUTHOR CONTRIBUTIONS

A.J.P., M.C.O., and M.J.O. led the study and interpreted the findings. A.J.P. collated gene sets with the assistance of J.H., D.H.K., and P.H. A.J.P. designed and implemented the analytic approach, with P.H. providing guidance on correction for multiple testing. J.T.R.W. led recruitment of the CLOZUK sample. E.R. and G.K. were responsible for all CNV calling in all samples. K.D.C., J.L.M., and S.A.M. coordinated the genotyping of the CLOZUK sample. A.J.P., assisted by M.C.O. and M.J.O., wrote the first draft of the manuscript, which was then commented on by other authors.

ACKNOWLEDGMENTS

For the CLOZUK sample we thank Novartis for its guidance and cooperation. We also thank staff at The Doctor's Laboratory, in particular Lisa Levett and Andrew Levett, for help and advice regarding sample acquisition. We acknowledge Kiran Mantripragada, Lesley Bates, Catherine Bresner, and Lucinda Hopkins for laboratory sample management.

The authors acknowledge the contribution of data from outside sources:

(a) Genetic Architecture of Smoking and Smoking Cessation accessed through dbGAP, study accession phs000404.v1.p1. Funding support for genotyping, which was performed at the Center for Inherited Disease Research (CIDR), was provided by 1 X01 HG005274-01 (CIDR is fully funded through a federal contract from the NIH to The Johns Hopkins University, contract number HHSN268200782096C). Assistance with genotype cleaning, as well as with general study coordination, was provided by the Gene Environment Association Studies (GENEVA) Coordinating Center (U01 HG004446). Funding support for collection of datasets and samples was provided by the Collaborative Genetic Study of Nicotine Dependence (COGEND; P01 CA089392) and the University of Wisconsin Transdisciplinary Tobacco Use Research Center (P50 DA019706, P50 CA084724).

(b) High Density SNP Association Analysis of Melanoma: Case-Control and Outcomes Investigation, dbGaP study accession phs000187.v1.p1. Research support to collect data and develop an application to support this project was provided by 3P50CA093459, 5P50CA097007, 5R01ES011740, and 5R01CA133996.

(c) Genetic Epidemiology of Refractive Error in the KORA (Kooperative Gesundheitsforschung in der Region Augsburg) Study, dbGaP study accession phs000303.v1.p1. Principal investigators: Dwight Stambolian, University of Pennsylvania, Philadelphia; H. Erich Wichmann, Institut für Humangenetik, Helmholtz-Zentrum München; National Eye Institute, NIH, Bethesda. Funded by R01 EY020483, NIH, Bethesda.

The work at Cardiff University was funded by Medical Research Council (MRC) Centre (G0800509) and Program Grants (G0801418) and the European Community's Seventh Framework Programme (HEALTH-F2-2010-241909, Project EU-GEI) and an MRC PhD Studentship to E.R. The schizophrenia samples were genotyped at the Broad Institute, and funded by a philanthropic gift to the Stanley Center for Psychiatric Research.

Received: November 28, 2014
Revised: March 26, 2015
Accepted: April 22, 2015
Published: June 3, 2015

REFERENCES

Ashburner, M., Ball, C.A., Blake, J.A., Botstein, D., Butler, H., Cherry, J.M., Davis, A.P., Dolinski, K., Dwight, S.S., Eppig, J.T., et al.; The Gene Ontology Consortium (2000). Gene ontology: tool for the unification of biology. Nat. Genet. *25*, 25–29.

Bartos, M., Vida, I., and Jonas, P. (2007). Synaptic mechanisms of synchronized gamma oscillations in inhibitory interneuron networks. Nat. Rev. Neurosci. *8*, 45–56.

Battaglia, A., Hoyme, H.E., Dallapiccola, B., Zackai, E., Hudgins, L., McDonald-McGinn, D., Bahi-Buisson, N., Romano, C., Williams, C.A., Brailey, L.L., et al. (2008). Further delineation of deletion 1p36 syndrome in 60 patients: a recognizable phenotype and common cause of developmental delay and mental retardation. Pediatrics *121*, 404–410.

Bauer, E.P., Schafe, G.E., and LeDoux, J.E. (2002). NMDA receptors and L-type voltage-gated calcium channels contribute to long-term potentiation and different components of fear memory formation in the lateral amygdala. J. Neurosci. *22*, 5239–5249.

Blake, J.A., Bult, C.J., Eppig, J.T., Kadin, J.A., and Richardson, J.E.; Mouse Genome Database Group (2014). The Mouse Genome Database: integration of and access to knowledge about the laboratory mouse. Nucleic Acids Res. *42*, D810–D817.

Buzsáki, G., and Draguhn, A. (2004). Neuronal oscillations in cortical networks. Science *304*, 1926–1929.

Buzsáki, G., and Watson, B.O. (2012). Brain rhythms and neural syntax: implications for efficient coding of cognitive content and neuropsychiatric disease. Dialogues Clin. Neurosci. *14*, 345–367.

Carcea, I., and Froemke, R.C. (2013). Cortical plasticity, excitatory-inhibitory balance, and sensory perception. Prog. Brain Res. *207*, 65–90.

Cardno, A.G., and Gottesman, I.I. (2000). Twin studies of schizophrenia: from bow-and-arrow concordances to star wars Mx and functional genomics. Am. J. Med. Genet. *97*, 12–17.

Carlsson, A. (1988). The current status of the dopamine hypothesis of schizophrenia. Neuropsychopharmacology *1*, 179–186.

Darnell, J.C., Van Driesche, S.J., Zhang, C., Hung, K.Y., Mele, A., Fraser, C.E., Stone, E.F., Chen, C., Fak, J.J., Chi, S.W., et al. (2011). FMRP stalls ribosomal translocation on mRNAs linked to synaptic function and autism. Cell *146*, 247–261.

Fernández, E., Collins, M.O., Uren, R.T., Kopanitsa, M.V., Komiyama, N.H., Croning, M.D., Zografos, L., Armstrong, J.D., Choudhary, J.S., and Grant, S.G. (2009). Targeted tandem affinity purification of PSD-95 recovers core postsynaptic complexes and schizophrenia susceptibility proteins. Mol. Syst. Biol. *5*, 269.

Friston, K.J. (1998). The disconnection hypothesis. Schizophr. Res. *30*, 115–125.

Fritschy, J.M., and Panzanelli, P. (2014). GABAA receptors and plasticity of inhibitory neurotransmission in the central nervous system. Eur. J. Neurosci. *39*, 1845–1865.

Fromer, M., Pocklington, A.J., Kavanagh, D.H., Williams, H.J., Dwyer, S., Gormley, P., Georgieva, L., Rees, E., Palta, P., Ruderfer, D.M., et al. (2014). De novo mutations in schizophrenia implicate synaptic networks. Nature *506*, 179–184.

Girard, S.L., Gauthier, J., Noreau, A., Xiong, L., Zhou, S., Jouan, L., Dionne-Laporte, A., Spiegelman, D., Henrion, E., Diallo, O., et al. (2011). Increased exonic de novo mutation rate in individuals with schizophrenia. Nat. Genet. *43*, 860–863.

Girirajan, S., Rosenfeld, J.A., Coe, B.P., Parikh, S., Friedman, N., Goldstein, A., Filipink, R.A., McConnell, J.S., Angle, B., Meschino, W.S., et al. (2012). Phenotypic heterogeneity of genomic disorders and rare copy-number variants. N. Engl. J. Med. *367*, 1321–1331.

Glessner, J.T., Reilly, M.P., Kim, C.E., Takahashi, N., Albano, A., Hou, C., Bradfield, J.P., Zhang, H., Sleiman, P.M., Flory, J.H., et al. (2010). Strong synaptic transmission impact by copy number variations in schizophrenia. Proc. Natl. Acad. Sci. USA *107*, 10584–10589.

Glykys, J., Mann, E.O., and Mody, I. (2008). Which GABA(A) receptor subunits are necessary for tonic inhibition in the hippocampus? J. Neurosci. *28*, 1421–1426.

Gulsuner, S., Walsh, T., Watts, A.C., Lee, M.K., Thornton, A.M., Casadei, S., Rippey, C., Shahin, H., Nimgaonkar, V.L., Go, R.C., et al.; Consortium on the

Genetics of Schizophrenia (COGS); PAARTNERS Study Group (2013). Spatial and temporal mapping of de novo mutations in schizophrenia to a fetal prefrontal cortical network. Cell *154*, 518–529.

Heller, E.A., Zhang, W., Selimi, F., Earnheart, J.C., Ślimak, M.A., Santos-Torres, J., Ibañez-Tallon, I., Aoki, C., Chait, B.T., and Heintz, N. (2012). The biochemical anatomy of cortical inhibitory synapses. PLoS ONE *7*, e39572.

Husi, H., and Grant, S.G. (2001). Isolation of 2000-kDa complexes of N-methyl-D-aspartate receptor and postsynaptic density 95 from mouse brain. J. Neurochem. *77*, 281–291.

Husi, H., Ward, M.A., Choudhary, J.S., Blackstock, W.P., and Grant, S.G. (2000). Proteomic analysis of NMDA receptor-adhesion protein signaling complexes. Nat. Neurosci. *3*, 661–669.

Inan, M., Petros, T.J., and Anderson, S.A. (2013). Losing your inhibition: linking cortical GABAergic interneurons to schizophrenia. Neurobiol. Dis. *53*, 36–48.

International Schizophrenia Consortium (2008). Rare chromosomal deletions and duplications increase risk of schizophrenia. Nature *455*, 237–241.

Iossifov, I., Ronemus, M., Levy, D., Wang, Z., Hakker, I., Rosenbaum, J., Yamrom, B., Lee, Y.H., Narzisi, G., Leotta, A., et al. (2012). De novo gene disruptions in children on the autistic spectrum. Neuron *74*, 285–299.

Joshi, S., and Kapur, J. (2013). N-methyl-D-aspartic acid receptor activation downregulates expression of δ subunit-containing GABAA receptors in cultured hippocampal neurons. Mol. Pharmacol. *84*, 1–11.

Kehrer, C., Maziashvili, N., Dugladze, T., and Gloveli, T. (2008). Altered excitatory-inhibitory balance in the NMDA-hypofunction model of schizophrenia. Front. Mol. Neurosci. *1*, 6.

Kirov, G., Gumus, D., Chen, W., Norton, N., Georgieva, L., Sari, M., O'Donovan, M.C., Erdogan, F., Owen, M.J., Ropers, H.H., and Ullmann, R. (2008). Comparative genome hybridization suggests a role for NRXN1 and APBA2 in schizophrenia. Hum. Mol. Genet. *17*, 458–465.

Kirov, G., Pocklington, A.J., Holmans, P., Ivanov, D., Ikeda, M., Ruderfer, D., Moran, J., Chambert, K., Toncheva, D., Georgieva, L., et al. (2012). De novo CNV analysis implicates specific abnormalities of postsynaptic signalling complexes in the pathogenesis of schizophrenia. Mol. Psychiatry *17*, 142–153.

Korn, J.M., Kuruvilla, F.G., McCarroll, S.A., Wysoker, A., Nemesh, J., Cawley, S., Hubbell, E., Veitch, J., Collins, P.J., Darvishi, K., et al. (2008). Integrated genotype calling and association analysis of SNPs, common copy number polymorphisms and rare CNVs. Nat. Genet. *40*, 1253–1260.

Laruelle, M., Frankle, W.G., Narendran, R., Kegeles, L.S., and Abi-Dargham, A. (2005). Mechanism of action of antipsychotic drugs: from dopamine D(2) receptor antagonism to glutamate NMDA facilitation. Clin. Ther. *27*, S16–S24.

Levinson, D.F., Duan, J., Oh, S., Wang, K., Sanders, A.R., Shi, J., Zhang, N., Mowry, B.J., Olincy, A., Amin, F., et al. (2011). Copy number variants in schizophrenia: confirmation of five previous findings and new evidence for 3q29 microdeletions and VIPR2 duplications. Am. J. Psychiatry *168*, 302–316.

Lewis, D.A., and Gonzalez-Burgos, G. (2006). Pathophysiologically based treatment interventions in schizophrenia. Nat. Med. *12*, 1016–1022.

Lewis, D.A., Curley, A.A., Glausier, J.R., and Volk, D.W. (2012). Cortical parvalbumin interneurons and cognitive dysfunction in schizophrenia. Trends Neurosci. *35*, 57–67.

Malhotra, D., and Sebat, J. (2012). CNVs: harbingers of a rare variant revolution in psychiatric genetics. Cell *148*, 1223–1241.

Malhotra, D., McCarthy, S., Michaelson, J.J., Vacic, V., Burdick, K.E., Yoon, S., Cichon, S., Corvin, A., Gary, S., Gershon, E.S., et al. (2011). High frequencies of de novo CNVs in bipolar disorder and schizophrenia. Neuron *72*, 951–963.

Mann, E.O., and Mody, I. (2010). Control of hippocampal gamma oscillation frequency by tonic inhibition and excitation of interneurons. Nat. Neurosci. *13*, 205–212.

Martin, L.J., Zurek, A.A., MacDonald, J.F., Roder, J.C., Jackson, M.F., and Orser, B.A. (2010). Alpha5GABAA receptor activity sets the threshold for long-term potentiation and constrains hippocampus-dependent memory. J. Neurosci. *30*, 5269–5282.

Miller, R. (1976). Schizophrenic psychology, associative learning and the role of forebrain dopamine. Med. Hypotheses *2*, 203–211.

Myles-Worsley, M., Tiobech, J., Browning, S.R., Korn, J., Goodman, S., Gentile, K., Melhem, N., Byerley, W., Faraone, S.V., and Middleton, F.A. (2013). Deletion at the SLC1A1 glutamate transporter gene co-segregates with schizophrenia and bipolar schizoaffective disorder in a 5-generation family. Am. J. Med. Genet. B. Neuropsychiatr. Genet. *162B*, 87–95.

O'Roak, B.J., Vives, L., Girirajan, S., Karakoc, E., Krumm, N., Coe, B.P., Levy, R., Ko, A., Lee, C., Smith, J.D., et al. (2012). Sporadic autism exomes reveal a highly interconnected protein network of de novo mutations. Nature *485*, 246–250.

Olney, J.W., and Farber, N.B. (1995). Glutamate receptor dysfunction and schizophrenia. Arch. Gen. Psychiatry *52*, 998–1007.

Pocklington, A.J., Cumiskey, M., Armstrong, J.D., and Grant, S.G. (2006). The proteomes of neurotransmitter receptor complexes form modular networks with distributed functionality underlying plasticity and behaviour. Mol. Syst. Biol. *2*, http://dx.doi.org/10.1038/msb4100041.

Purcell, S.M., Moran, J.L., Fromer, M., Ruderfer, D., Solovieff, N., Roussos, P., O'Dushlaine, C., Chambert, K., Bergen, S.E., Kähler, A., et al. (2014). A polygenic burden of rare disruptive mutations in schizophrenia. Nature *506*, 185–190.

Rauch, A., Wieczorek, D., Graf, E., Wieland, T., Endele, S., Schwarzmayr, T., Albrecht, B., Bartholdi, D., Beygo, J., Di Donato, N., et al. (2012). Range of genetic mutations associated with severe non-syndromic sporadic intellectual disability: an exome sequencing study. Lancet *380*, 1674–1682.

Raychaudhuri, S., Korn, J.M., McCarroll, S.A., Altshuler, D., Sklar, P., Purcell, S., and Daly, M.J.; International Schizophrenia Consortium (2010). Accurately assessing the risk of schizophrenia conferred by rare copy-number variation affecting genes with brain function. PLoS Genet. *6*, e1001097.

Rees, E., Walters, J.T., Chambert, K.D., O'Dushlaine, C., Szatkiewicz, J., Richards, A.L., Georgieva, L., Mahoney-Davies, G., Legge, S.E., Moran, J.L., et al.; Wellcome Trust Case Control Consortium (2014a). CNV analysis in a large schizophrenia sample implicates deletions at 16p12.1 and SLC1A1 and duplications at 1p36.33 and CGNL1. Hum. Mol. Genet. *23*, 1669–1676.

Rees, E., Walters, J.T., Georgieva, L., Isles, A.R., Chambert, K.D., Richards, A.L., Mahoney-Davies, G., Legge, S.E., Moran, J.L., McCarroll, S.A., et al. (2014b). Analysis of copy number variations at 15 schizophrenia-associated loci. Br. J. Psychiatry *204*, 108–114.

Roberts, E. (1972). Prospects for research on schizophrenia. An hypotheses suggesting that there is a defect in the GABA system in schizophrenia. Neurosci. Res. Program Bull. *10*, 468–482.

Rodrigues, S.M., Schafe, G.E., and LeDoux, J.E. (2001). Intra-amygdala blockade of the NR2B subunit of the NMDA receptor disrupts the acquisition but not the expression of fear conditioning. J. Neurosci. *21*, 6889–6896.

Sanders, S.J., Murtha, M.T., Gupta, A.R., Murdoch, J.D., Raubeson, M.J., Willsey, A.J., Ercan-Sencicek, A.G., DiLullo, N.M., Parikshak, N.N., Stein, J.L., et al. (2012). De novo mutations revealed by whole-exome sequencing are strongly associated with autism. Nature *485*, 237–241.

Schizophrenia Working Group of the Psychiatric Genomics Consortium (2014). Biological insights from 108 schizophrenia-associated genetic loci. Nature *511*, 421–427.

Shapira, S.K., McCaskill, C., Northrup, H., Spikes, A.S., Elder, F.F., Sutton, V.R., Korenberg, J.R., Greenberg, F., and Shaffer, L.G. (1997). Chromosome 1p36 deletions: the clinical phenotype and molecular characterization of a common newly delineated syndrome. Am. J. Hum. Genet. *61*, 642–650.

Stephan, K.E., Friston, K.J., and Frith, C.D. (2009). Dysconnection in schizophrenia: from abnormal synaptic plasticity to failures of self-monitoring. Schizophr. Bull. *35*, 509–527.

Sullivan, P.F., Daly, M.J., and O'Donovan, M. (2012). Genetic architectures of psychiatric disorders: the emerging picture and its implications. Nat. Rev. Genet. *13*, 537–551.

Szatkiewicz, J.P., O'Dushlaine, C., Chen, G., Chambert, K., Moran, J.L., Neale, B.M., Fromer, M., Ruderfer, D., Akterin, S., Bergen, S.E., et al. (2014). Copy number variation in schizophrenia in Sweden. Mol. Psychiatry *19*, 762–773.

Uhlhaas, P.J., and Singer, W. (2010). Abnormal neural oscillations and synchrony in schizophrenia. Nat. Rev. Neurosci. *11*, 100–113.

Walsh, T., McClellan, J.M., McCarthy, S.E., Addington, A.M., Pierce, S.B., Cooper, G.M., Nord, A.S., Kusenda, M., Malhotra, D., Bhandari, A., et al. (2008). Rare structural variants disrupt multiple genes in neurodevelopmental pathways in schizophrenia. Science *320*, 539–543.

Wang, K., Li, M., Hadley, D., Liu, R., Glessner, J., Grant, S.F., Hakonarson, H., and Bucan, M. (2007). PennCNV: an integrated hidden Markov model designed for high-resolution copy number variation detection in whole-genome SNP genotyping data. Genome Res. *17*, 1665–1674.

Williams, N.M., Zaharieva, I., Martin, A., Langley, K., Mantripragada, K., Fossdal, R., Stefansson, H., Stefansson, K., Magnusson, P., Gudmundsson, O.O., et al. (2010). Rare chromosomal deletions and duplications in attention-deficit hyperactivity disorder: a genome-wide analysis. Lancet *376*, 1401–1408.

Windpassinger, C., Kroisel, P.M., Wagner, K., and Petek, E. (2002). The human gamma-aminobutyric acid A receptor delta (GABRD) gene: molecular characterisation and tissue-specific expression. Gene *292*, 25–31.

Xu, B., Roos, J.L., Levy, S., van Rensburg, E.J., Gogos, J.A., and Karayiorgou, M. (2008). Strong association of de novo copy number mutations with sporadic schizophrenia. Nat. Genet. *40*, 880–885.

Xu, B., Ionita-Laza, I., Roos, J.L., Boone, B., Woodrick, S., Sun, Y., Levy, S., Gogos, J.A., and Karayiorgou, M. (2012). De novo gene mutations highlight patterns of genetic and neural complexity in schizophrenia. Nat. Genet. *44*, 1365–1369.

Yizhar, O., Fenno, L.E., Prigge, M., Schneider, F., Davidson, T.J., O'Shea, D.J., Sohal, V.S., Goshen, I., Finkelstein, J., Paz, J.T., et al. (2011). Neocortical excitation/inhibition balance in information processing and social dysfunction. Nature *477*, 171–178.

Neuron

Supplemental Information

Novel Findings from CNVs Implicate Inhibitory and Excitatory Signaling Complexes in Schizophrenia

Andrew J. Pocklington, Elliott Rees, James T.R. Walters, Jun Han, David H. Kavanagh, Kimberly D. Chambert, Peter Holmans, Jennifer L. Moran, Steven A. McCarroll, George Kirov, Michael C. O'Donovan, and Michael J. Owen

Supplemental Information

1. Supplemental Data

Table S1: CNS gene set association, Related to Tables 1-4

Table S2: Known schizophrenia loci, Related to Tables 1-4

Table S3: Enriched CNS gene-sets, known loci removed, Related to Tables 2-4

Table S4: Enriched CNS gene sets, single-gene association (combined), Related to Results

Table S5: Enriched CNS gene sets, single-gene association (deletions), Related to Results

Table S6: Enriched CNS gene sets, single-gene association (duplications), Related to Results

Table S7: MGI gene set association, conditional analysis, Related to Results

Table S8: GO gene set association, conditional analysis, Related to Results

Table S9: Associated CNS gene sets - overlap with NS de novo rare variants, Related to Table 5

Table S10: GABA$_A$ receptor complex, single-gene enrichment (complete), Related to Discussion

2. Supplemental Experimental Procedures

Samples, genotyping and CNV quality control

CLOZUK

Molecular Genetics of Schizophrenia (MGS)

International Schizophrenia Consortium (ISC)

Additional CNV QC for CLOZUK, ISC and MGS

Validation of CLOZUK

Gene annotations

Gene set enrichment test

Enrichment beyond CNS-related terms

Identification of 'minimal set' capturing association signal in enriched CNS terms

Removing signal from known loci

Calculation of gene set odds ratios

CNV size and number of genes hit as predictors of case-control status

Correction for multiple testing

3. Supplemental References

Supplemental Data

Legends

Table S1: CNS gene set association, Related to Tables 1-4

Association results for all 134 CNS gene sets tested in the combined analysis of deletions and duplications together and the analysis of deletions or duplications separately. Uncorrected (P) and Bonferroni corrected (P adjusted) one-sided p-values for enrichment in case CNVs are given, together with the source of the gene set and the number of autosomal genes in each set (N gene). As an additional test exploring the sensitivity of our results to CNV calling, we repeated our analysis of CNS-related gene sets restricting to CNVs > 500kb where we can expect very high concordance between chips. Of the 28 associations we report with a corrected P < 0.05, only 1 was not nominally associated in CNVs >500kb.

Table S2: Known schizophrenia loci, Related to Tables 1-4

Confirmed schizophrenia loci were taken from the largest systematic survey to date (Rees et al., 2014b). For each locus we list its position (Position (Mb, hg19)) together with the number (N) and percentage of individuals carrying the CNV (rate (%)) for cases and controls from

each of the three contributing studies. The total number/rate of these 11 loci in cases and controls is also given for each study. In this table only CNVs spanning an entire locus are counted; for analyses involving the removal of known loci, all CNVs overlapping these loci are removed (see Supplemental Experimental Procedures).

Table S3: Enriched CNS gene-sets, known loci removed, Related to Tables 2-4

Initial columns summarise the association data for CNS gene sets with Bonferroni corrected p-value < 0.05 in each of the three analyses: deletions and duplications being analysed together (Combined) or separately. The final two columns give the sign of the regression coefficient and uncorrected p-value for each gene set when CNVs overlapping well-supported schizophrenia loci were removed. Gene sets with $P_{uncorrected} < 0.05$ after removal of known loci are highlighted in bold.

Table S4: Enriched CNS gene sets, single-gene association (combined), Related to Results

For each CNS gene set with Bonferroni corrected p-value < 0.05 in the analysis of deletions and duplications combined, this table lists those genes with an uncorrected single-gene association p-value < 0.05 (again, in a combined analysis of deletions and duplications together). In addition to gene identifiers and chromosomal locations, the table lists the number of case and control CNVs that overlap the gene (N case and N ctrl respectively), raw and Bonferroni corrected p-values (P, P adjusted), and whether the gene is found in a well-supported schizophrenia CNV locus (known locus, locus type). Bonferroni correction is for the total number of single gene tests. Genes lying outside the boundaries of a known CNV locus, but whose association signal was clearly driven by that locus, were annotated as lying in 'xxx (extended)', where xxx is the corresponding locus.

Table S5: Enriched CNS gene sets, single-gene association (deletions), Related to Results

For each CNS gene set with Bonferroni corrected p-value < 0.05 in the analysis of deletions, this table lists those genes with an uncorrected single-gene association p-value < 0.05 (again, in the analysis of deletions alone). Columns are identical to those found in Table S4.

Table S6: Enriched CNS gene sets, single-gene association (duplications), Related to Results

For each CNS gene set with Bonferroni corrected p-value < 0.05 in the analysis of duplications, this table lists those genes with an uncorrected single-gene association p-value < 0.05 (again, in the analysis of duplications alone). Columns are identical to those found in Table S4.

Table S7: MGI gene set association, conditional analysis, Related to Results

Association results for all MGI gene sets tested in the combined analysis of deletions and duplications together and the analysis of deletions or duplications separately. Uncorrected (P) and Bonferroni corrected (P adjusted) one-sided conditional p-values for enrichment in case CNVs are given, together with the gene set name, id and number of autosomal genes (N gene).

Table S8: GO gene set association, conditional analysis, Related to Results

Association results for all GO gene sets tested in the combined analysis of deletions and duplications together and the analysis of deletions or duplications separately. Uncorrected (P) and Bonferroni corrected (P adjusted) one-sided conditional p-values for enrichment in case CNVs are given, together with the gene set name, id and number of autosomal genes in each set (N gene).

Table S9: Associated CNS gene sets - overlap with NS de novo rare variants, Related to Table 5

For each gene set with a Bonferroni corrected P < 0.05 identified by our analyses (Tables 2-4), we investigated enrichment for non-synonymous (NS) *de novo* rare variants from individuals with schizophrenia. Here we list the number of genes in each gene set (N gene); the number of de novo rare variants found within these genes (N observed); the number of variants expected (N expected); plus uncorrected (P) and Bonferroni corrected (P adjusted) p-values. For comparison, this analysis was then repeated using NS *de novo* rare variants identified in controls, with exactly the same correction procedure. See Experimental Procedures for the source of variants used.

Table S10: $GABA_A$ receptor complex, single-gene enrichment (complete), Related to Discussion

Single-gene CNV counts and enrichment p-values for all genes in the GABA receptor complex gene set. Genes found on the X chromosome, which was not analysed here, are listed for completeness. Columns are as given in Tables S4-S6.

Supplemental Tables

Table S3:

	gene set	N_{gene}	before removal coeff	before removal P	after removal coeff	after removal P
Combined	**NMDAR network**	**59**	**+**	**$4.3x10^{-9}$**	**+**	**$1.0x10^{-6}$**
	$GABA_A$	15	+	$3.0x10^{-6}$	+	0.075
	**abnormal associative learning**	**193**	**+**	**$1.6x10^{-5}$**	**+**	**0.0071**
	**abnormal long term potentiation**	**145**	**+**	**$2.0x10^{-5}$**	**+**	**0.031**
	**abnormal behavior**	**1973**	**+**	**$5.1x10^{-5}$**	**+**	**0.0025**
	**abnormal CNS synaptic transmission**	**371**	**+**	**$5.5x10^{-5}$**	**+**	**0.015**
Deletion	**PSD-95 (core)**	**58**	**+**	**$4.3x10^{-11}$**	**+**	**0.0022**
	**abnormal neural plate morphology**	**23**	**+**	**$2.1x10^{-7}$**	**+**	**0.0097**
	abnormal prepulse inhibition	74	+	$3.3x10^{-7}$	-	0.53
	**abnormal behavior**	**1973**	**+**	**$3.0x10^{-6}$**	**+**	**0.015**
	**abnormal fear/anxiety-related behavior**	**216**	**+**	**$3.2x10^{-6}$**	**+**	**0.012**
	abnormal CNS synaptic transmission	371	+	$5.1x10^{-6}$	+	0.29
	abnormal spatial working memory	38	+	$5.6x10^{-6}$	+	0.13
	abnormal synaptic transmission	437	+	$1.1x10^{-5}$	+	0.14
	abnormal emotion/affect behavior	369	+	$1.1x10^{-5}$	+	0.083
	**abnormal neuron differentiation**	**206**	**+**	**$2.8x10^{-5}$**	**+**	**0.042**
	abnormal spatial learning	156	+	$4.8x10^{-5}$	+	0.089
	abnormal social/conspecific interaction	243	+	$4.8x10^{-5}$	-	0.61
	abnormal learning/memory/conditioning	424	+	$7.3x10^{-5}$	+	0.33
	abnormal miniature excitatory postsynaptic currents	62	+	0.00010	+	0.091
Duplication	**abnormal associative learning**	**193**	**+**	**$1.6x10^{-10}$**	**+**	**0.0017**
	**NMDAR network**	**59**	**+**	**$2.5x10^{-9}$**	**+**	**0.00066**
	abnormal long term potentiation	145	+	$1.1x10^{-6}$	+	0.27
	abnormal avoidance learning behavior	56	+	$1.6x10^{-6}$	+	0.10
	**abnormal cued conditioning behavior**	**68**	**+**	**$1.4x10^{-5}$**	**+**	**0.00060**
	**$GABA_A$**	**15**	**+**	**$5.4x10^{-5}$**	**+**	**0.043**
	**abnormal contextual conditioning behavior**	**89**	**+**	**0.00011**	**+**	**0.016**

Supplemental Experimental Procedures

Samples, genotyping and CNV quality control

Tables listing genotyping chips, number of probes and number of samples post QC for each of the 3 studies used in this analysis are given below. The CLOZUK study drew together samples genotyped on a range of Illumina chips, control samples being chosen to ensure chips were as similar to those for cases as possible. Given that different Illumina chips were used in the CLOZUK sample, only probes present on all of these chips (N=520,766) were used to call CNVs, ensuring that all CNVs called on one chip were capable of being called on the others.

CLOZUK

A full description and ascertainment of the CLOZUK cases is given in (Rees, Walters et al. 2014, PMID: 24163246). Briefly, the case sample utilised here consists of patients taking clozapine. Blood was obtained from these patients through collaboration with Novartis, the manufacturer of a proprietary form of clozapine (Clozaril). These patients were aged 18-90 and had received a recorded diagnosis of treatment resistant schizophrenia. In the UK, treatment resistant schizophrenia implies a lack of satisfactory clinical improvement to adequate trials of at least two other antipsychotics. We excluded those with diagnoses other than treatment resistant schizophrenia and those prescribed clozapine for off-license indications. All cases were genotyped on either Illumina HumanOmniExpress-12v1 or Illumina HumanOmniExpressExome-8v.1 arrays.

The CLOZUK control sample has been described previously (Rees et al., 2014). This sample consisted of four non-psychiatric control datasets obtained from either the Database of Genotypes and Phenotypes (dbGaP) or the European Genome-Phenome Archive (EGA).

CLOZUK	Source (accession ID)	Array (N probes)	N common Illumina probes used to Call CNVs	N samples post QC, Europeans only
SZ Batch 1	Broad Institute	HumanOmniExpress-12v1 (730,525)	520,766	2,148
SZ Batch 2	Broad Institute	HumanOmniExpressExome-8v1 (951,117)	520,766	3,205
SZ Batch 3	Broad Institute	HumanOmniExpressExome-8v1 (951,117)	520,766	392
The Genetic Architecture of **Smoking** and Smoking Cessation	dbGaP (phs000404.v1.p1)	Illumina HumanOmni2.5 (2,443,179)	520,766	938
High Density SNP Association Analysis of **Melanoma**: Case-Control and Outcomes Investigation	dbGaP (phs000187.v1.p1)	Illumina HumanOmni1_Quad_v1-0-B (1,051,295)	520,766	2,955
Genetic Epidemiology of Refractive Error in the **KORA** Study	dbGaP (phs000303.v1.p1)	Illumina HumanOmni2.5 (2,443,179)	520,766	1,857
**WTCCC2** project samples from National Blood Donors (NBS) Cohort	EGA (EGAD00000000024)	Illumina 1.2M (1,238,733)	520,766	2,363
**WTCCC2** project samples from 1958 British Birth Cohort	EGA (EGAD00000000022)	Illumina 1.2M (1,238,733)	520,766	2,562

These four datasets were derived from a study on smoking and smoking cessation (dbGaP phs000404.v1.p1), melanoma (dbGaP phs000187.v1.p1), refractive error (dbGaP phs000303.v1.p1) and WTCCC2 (EGA EGAD00000000024 and EGAD00000000022), which combined amount to 12,080 samples before QC. These were genotyped on Illumina HumanOmni2.5, Illumina HumanOmni1_Quad_v1-0-B, Illumina HumanOmni2.5 and Illumina 1.2M arrays respectively (see table above).

Principal component analysis (PCA) was performed to derive the ancestries of the CLOZUK cases and controls by combining the data with Hapmap genotypes. Samples were stratified

into those from a European, African or ‘other’ origin. In this paper we only included those of European origin. Further details can be found in (Rees et al., 2014a).

Raw intensity data from each case/control dataset (listed in the table above) were independently processed and analysed to account for potential batch effects. The PennCNV (Wang et al., 2007) algorithm with GC correction was used to detect CNVs from the 520,766 probes common to all Illumina arrays used to genotype the CLOZUK sample. Samples were subjected to rigorous QC and excluded if for any one of the following metrics they represented an outlier in their source dataset: Log R ratio standard deviation, B-allele frequency drift, wave factor and total number of CNVs called per person.

Molecular Genetics of Schizophrenia (MGS)

Details of the MGS cohort have been described elsewhere (Levinson et al., 2011). Our CNV analysis and QC of this sample has also been described previously (Rees et al., 2014a). Briefly, the samples were genotyped at the Broad Institute, Cambridge, Massachusetts, using Affymetrix 6.0 genotyping arrays. All schizophrenic patients met DSM-IV criteria for schizophrenia or schizoaffective disorder. CNVs were called using the Birdsuite algorithm (Korn et al., 2008).

MGS	Array (N probes)	N samples post QC, Europeans only
SZ cases	Affymetrix 6.0 (1,854,910)	2,215
Controls	Affymetrix 6.0 (1,854,910)	2,556

International Schizophrenia Consortium (ISC)

Details of the ISC sample have been described elsewhere (International Schizophrenia Consortium, 2008). The sample was genotyped at the Broad Institute, Cambridge,

Massachusetts, using Affymetrix 6.0 or Affymetrix 5.0 genotyping arrays and consists of six European populations. CNVs were called using the Birdsuite algorithm (Korn et al., 2008).

ISC	Array (N probes)	N samples post QC, Europeans only
SZ cases	Affymetrix 6.0 (1,854,910)	1,583
Controls	Affymetrix 6.0 (1,854,910)	2,095
SZ cases	Affymetrix 5.0 (440,638)	1,812
Controls	Affymetrix 5.0 (440,638)	1,090

Additional CNV QC for CLOZUK, ISC and MGS

For individuals/CNVs passing QC procedures performed by the original studies, CNV calls were joined if the distance separating them was less than 50% of their combined length. CNV calls were excluded if they overlapped with low copy repeats by more than 50% of their length, or had a probe density (calculated by dividing the size of the CNV by the number of probes covering it) less than 1 probe/20kb. We used PLINK (Purcell et al., 2007) to remove CNVs with a frequency > 1% in their respective sample (CLOZUK, ISC or MGS). We then applied an *in silico* median Z-score outlier method of CNV validation, described in detail elsewhere (Kirov et al., 2012; Rees et al., 2014), to all remaining CNVs. This method has been shown to be effective for the removal of false positive CNV calls and detecting CNVs missed by calling (Kirov et al., 2012). We did not perform Z-score validation for the ISC study as we did not have access to the raw intensity data.

Following QC (performed separately for each study), protein-coding genes overlapping CNVs were identified using genomic locations for the appropriate build of the human genome: Build 35 of the human genome for ISC, Build 36 for MGS and Build 37 for CLOZUK. Studies were then collated and CNVs <100kb in size and/or covered by < 15 probes removed prior to analysis. Non-European individuals were removed prior to analysis, leaving 5,745 cases and 10,675 controls in CLOZUK; 2,215 cases and 2,556 controls in

MGS; and 3,395 cases and 3,185 controls in ISC. The number of CLOZUK cases used in the current study differs from that reported in (Rees et al., 2014a) as that study included an additional 571 cases from the CardiffCOGs sample.

In performing gene set enrichment analyses we specifically included covariates for genotyping chip and study to remove any biases due to differences between Affymetrix 5.0, Affymetrix 6.0 and Illumina arrays and between the cohorts used in each individual study. We would add that since matched sets of cases and controls were genotyped on each Affymetrix array, the use of multiple chips in ISC and MGS does not cause an increase in false positives. To investigate whether batch effects in CLOZUK (due to the multiple sources of controls) were driving our results, we took all CNS-related gene sets with a Bonferroni corrected p-value < 0.05 and tested for significant differences in CNV overlap between controls genotyped in different studies or on different chips. This was performed using the same logistic regression model as the case-control CNV enrichment test from our primary analysis, but with 'cases' now being control CNVs from one study/chip and 'controls' being control CNVs from a different chip/study (and obviously using no covariates for chip or study). Calculating two-sided p-values for all potential chip-chip and control study-control study pairings, there were no significant differences after correcting for the number of comparisons made (data not shown).

Validation of CLOZUK:

Validation of Clinical Diagnosis

We used the Cardiff Cognition in Schizophrenia (Cardiff COGS) sample to assess the validity of a psychiatrist-assigned diagnosis of treatment resistant schizophrenia as applied in CLOZUK. The Cardiff COGS sample is a conventional sample of those with schizophrenia

recruited via secondary care, mainly outpatient, mental health services in Wales and England. The recruitment procedures included inviting patients from clozapine clinics, irrespective of diagnosis. Consenting participants were interviewed with the Schedules for Clinical Assessment in Neuropsychiatry (SCAN) (Wing et al., 1990) and consensus research diagnoses were agreed with reference to the interview and clinical notes according to DSM-IV criteria.

Validation Procedure

Prior to the research interview we obtained clinicians' diagnoses for all participants in Cardiff COGS. From participants on clozapine we selected those with a clinical diagnosis of schizophrenia and confirmed that this matched the diagnosis provided when the participant was started on clozapine (i.e. treatment resistant schizophrenia) so as to be equivalent to the samples identified as having schizophrenia in CLOZUK. We then compared this diagnosis with the consensus research DSM-IV diagnosis.

Results

We identified 214 participants within CardiffCOGS (n=905) who were taking clozapine and had a clinician-assigned diagnosis of treatment resistant schizophrenia. Following consensus research diagnosis, 194 of these participants were identified as having DSMIV schizophrenia or schizoaffective disorder depressed sub-type, giving a positive predictive value (PPV) of 90.7%.

Many international groups and consortia also consider other diagnoses as 'schizophrenia' samples, namely schizoaffective disorder bipolar type, delusional disorder and schizophreniform disorders (Schizophrenia Working Group of the Psychiatric Genomics Consortium, 2014). If we expand our analysis to include these categories then 210 of 214

(PPV=98.1%) of those on clozapine with a clinical diagnosis of schizophrenia would receive a DSMIV research diagnosis of one of these schizophrenia spectrum disorders.

These results are entirely consistent with equivalent reports of the validity of clinician diagnoses in two Scandanavian studies (Ekholm et al., 2005; Jakobsen et al., 2005).

Molecular/Genetic Validation

In the largest GWAS meta-analysis to date, the schizophrenia working group of the Psychiatric Genomics Consortium identified 40 target subgroups within their primary GWAS analysis and performed a leave-one-out analysis (Schizophrenia Working Group of the Psychiatric Genomics Consortium, 2014). Using risk alleles identified in the remainder of the primary sample, polygenic risk profile scores were calculated for all individuals in the target subgroup; the ability of these scores to distinguish between cases and controls was then evaluated. The predictive value of the risk profile score when applied to CLOZUK was indistinguishable from its performance in other schizophrenia subgroups, indeed the values for R2 (on the liability scale) for CLOZUK are the 5th highest of all subsamples, implying that CLOZUK is one of the samples most highly enriched for schizophrenia risk alleles (see data for 'noclo_clo' in Extended data Figure 6b from (Schizophrenia Working Group of the Psychiatric Genomics Consortium, 2014)). In terms of CNVs, the rate of individual confirmed schizophrenia loci in CLOZUK are entirely consistent with those of the other schizophrenia studies (Table S2 of this paper).

Taken together, the clinical and molecular evidence strongly validate CLOZUK as a schizophrenia sample.

Gene annotations

The ARC and NMDAR network gene sets used here were taken from (Kirov et al., 2012); the $GABA_A$ receptor complex gene set is listed in Table S10. All other gene sets are available from the authors upon request.

GO

Gene Ontology (GO) annotations were taken from NCBI gene2go (ftp://ftp.ncbi.nih.gov/DATA), using Homo Sapiens annotations only. Parent terms were identified for each GO term through the AmiGO ontology (http://www.geneontology.org/GO.downloads.ontology.shtml). We used "is_a" and "part_of" (but not "regulates") to define child-parent relationships between terms. The parent terms of each GO term assigned to a gene in gene2go were also assigned to that gene. When performing enrichment analyses we restricted to GO terms containing between 20 and 2000 autosomal genes, a total of 4026 terms.

MGI

The Mammalian Phenotype (MP) ontology and gene annotations were downloaded from the Mouse Genome Database (Blake et al., 2011) within the Mouse Genome Informatics (MGI) online resource (http://www.informatics.jax.org). Gene annotations arising from transgene and multi-gene manipulations were removed. Parent terms were identified for each MP term and assigned to all genes annotated with that child term. Genes were mapped to human using file HOM_MouseHumanSequence.rpt, also downloaded from MGI. Within this file human and mouse genes are organised into orthologous groups identified by HomoloGene id. To ensure the unambiguous annotation of human genes, we discarded all phenotypic information from mouse genes with non-unique (1-many, many-1, many-many) orthology relationships (i.e. HomoloGene groups containing multiple mouse and/or human gene ids).

When performing enrichment analyses we restricted to MGI terms with more than 20 autosomal genes, a total of 2616 terms (of which 118 were extracted for use as CNS-related gene sets). As MGI terms relate to specific biological processes we felt there was no need to place an upper bound on gene set size, used above to remove extremely large, generic GO annotations.

Gene set enrichment test

For each gene set, the number of genes ‘hit’ by case and control CNVs were compared; a gene was counted as being hit by a CNV if the CNV overlapped any part of its length. To overcome biases related to gene and CNV size, and to control for differences between studies and genotyping chips, the following logistic regression models were fitted to the combined set of CNVs:

(a) logit (pr(case)) = study + chip + CNV size + total number of genes hit

(b) logit (pr(case)) = study + chip + CNV size + total number of genes hit + number of genes hit in gene set

Comparing the change in deviance between models (a) and (b), a one-sided test for an excess of genes in the gene set being hit by case CNVs was performed.

By comparing case to control CNVs, this analysis allows for the possibility of non-random CNV location unrelated to disease (i.e. CNVs tend to occur in specific locations of the genome and this is unrelated to case status, both in cases and controls). The inclusion of CNV size in the regression allows for the fact that case CNVs are larger than control CNVs

(and thus likely to hit more genes, regardless of function), even when restricting to those >100kb in length (see Results). Inclusion of the total number of genes hit in the regression corrects for case CNVs hitting more genes overall (regardless of function) than control CNVs. It should also be noted that since we compare between cases and controls, gene size (which is the same in cases and controls) is not a source of potential bias: CNVs of given size have exactly the same chance of overlapping a particular gene in both cases and controls.

Since case and control samples from the CLOZUK study were genotyped on different chips, we were unable to completely control for possible inter-chip differences. This is unlikely to influence our analyses: calling is most robust for large CNVs; calling was restricted to probes present on all arrays; and the arrays used were in any case comparable in coverage (Rees et al., 2014a). The chip covariate therefore took the values 'Affymetrix 5.0' (subset of ISC samples), 'Affymetrix 6.0' (subset of ISC and all MGS samples) and 'Illumina' (all CLOZUK samples). As a further check we took all CNS-related gene sets with a Bonferroni corrected p-value < 0.05 and tested for differences in CNV overlap between controls genotyped in different studies or on different chips (see 'Samples, genotyping and CNV quality control: Additional QC' above); no significant differences were found.

Enrichment beyond CNS-related terms

To determine whether any GO or MGI annotation showed evidence for enrichment in case CNVs that was independent of the association signal captured by CNS-related gene sets, the following regression models were fitted.

(a) logit (pr(case)) = study + chip + CNV size + total number of genes hit + CNS terms

(b) logit (pr(case)) = study + chip + CNV size + total number of genes hit + CNS terms + number of genes hit in gene set (from GO or MGI)

where CNS terms = number of genes hit in CNS gene set X + number of genes hit in CNS gene set Y + …

These were constructed by adding a subset of CNS terms, capturing the enrichment signal arising from all CNS gene sets with $P_{corrected} < 0.05$, to the regression models described earlier (see 'Enrichment test' above). The identification of this CNS subset is described in the main text and in greater detail below, its sole purpose being to minimise the number of additional model parameters to be fitted (i.e. compared to adding all CNS terms with $P_{corrected} < 0.05$). Comparing the change in deviance between models (a) and (b), a one-sided test for an excess of genes in the GO or MGI gene set being hit by case CNVs was performed.

Identification of 'minimal set' capturing association signal in enriched CNS terms

To capture the enrichment signal arising from CNS gene sets with $P_{corrected} < 0.05$, we added the most significant such term as a covariate to the regression model and recalculated gene set enrichment for each of the remaining terms. The term with the most significant residual enrichment was then added to the model and the process repeated until there was no residual association ($P_{uncorrected} < 0.05$) in the remaining terms. This iterative procedure is captured in the tables below. Initial columns (up to P) summarise the association data for CNS gene sets with Bonferroni $P_{corrected} < 0.05$. The remaining columns identify terms successively added to the original regression model and list residual enrichment p-values for the resulting extended model. The most significant p-value at each stage of the analysis (identifying the next term to be added to the model) is highlighted in bold.

For example, in the combined analysis of deletions and duplications the most significantly associated gene set was the NMDAR network gene set (see column 'P' in 'Combined' table below). With this is included as an extra covariate, the original regression model now becomes:

(a) logit (pr(case)) = study + chip + CNV size + total number of genes hit + number of NMDAR genes hit

To find the residual enrichment of the remaining gene sets (column 'NMDAR network' in 'Combined' table below), we compare the change in deviance between models (a) and (b):

(b) logit (pr(case)) = study + chip + CNV size + total number of genes hit + number of NMDAR genes hit **+ number of genes hit in gene set**

performing a one-sided test for an excess of genes in the gene set being hit by case CNVs. The $GABA_A$ gene set, which has the most significant residual association, is then added to (a) and the process repeated until no term has a residual $P_{uncorrected} < 0.05$ (see final column in 'Combined' table below).

Combined:

	N_{gene}	P	NMDAR network	$GABA_A$	abnormal behavior
NMDAR network	59	**4.3×10^{-9}**	1	1	1
$GABA_A$	15	3.0×10^{-6}	**7.1×10^{-6}**	1	1
abnormal associative learning	193	1.6×10^{-5}	0.0060	0.028	0.088
abnormal long term potentiation	145	2.0×10^{-5}	0.0054	0.020	0.091
abnormal behavior	1973	5.1×10^{-5}	0.00032	**0.0052**	1
abnormal CNS synaptic transmission	371	5.5×10^{-5}	0.0016	0.020	0.24

Deletion:

	N_{gene}	P	PSD-95 (core)	abnormal fear/anxiety-related behavior	abnormal neural plate morphology
PSD-95 (core)	58	**$4.3x10^{-11}$**	1	1	1
abnormal neural plate morphology	23	$2.1x10^{-7}$	0.00015	**0.0020**	1
abnormal prepulse inhibition	74	$3.3x10^{-7}$	0.034	0.27	0.67
abnormal behavior	1973	$3.0x10^{-6}$	0.013	0.40	0.44
abnormal fear/anxiety-related behavior	216	$3.2x10^{-6}$	**0.00015**	1	1
abnormal CNS synaptic transmission	371	$5.1x10^{-6}$	0.090	0.50	0.67
abnormal spatial working memory	38	$5.6x10^{-6}$	0.0029	0.043	0.18
abnormal synaptic transmission	437	$1.1x10^{-5}$	0.060	0.79	0.85
abnormal emotion/affect behavior	369	$1.1x10^{-5}$	0.0016	0.72	0.94
abnormal neuron differentiation	206	$2.8x10^{-5}$	0.0046	0.026	0.052
abnormal spatial learning	156	$4.8x10^{-5}$	0.0018	0.26	0.35
abnormal social/conspecific interaction	243	$4.8x10^{-5}$	0.16	0.66	0.89
abnormal learning/memory/conditioning	424	$7.3x10^{-5}$	0.055	0.90	0.93
abnormal miniature excitatory postsynaptic currents	62	0.00010	0.58	0.45	0.36

Duplication:

	N_{gene}	P	abnormal associative learning	NMDAR network	$GABA_A$
abnormal associative learning	193	**$1.6x10^{-10}$**	1	1	1
NMDAR network	59	$2.5x10^{-9}$	**$2.7x10^{-5}$**	1	1
abnormal long term potentiation	145	$1.1x10^{-6}$	0.15	0.33	0.42
abnormal avoidance learning behavior	56	$1.6x10^{-6}$	0.18	0.38	0.20
abnormal cued conditioning behavior	68	$1.4x10^{-5}$	0.20	0.12	0.25
$GABA_A$	15	$5.4x10^{-5}$	0.0051	**0.0047**	1
abnormal contextual conditioning behavior	89	0.00011	0.69	0.47	0.75

Removing signal from known loci

To investigate whether gene set enrichment was solely driven by CNVs at loci well supported by current data, we removed all CNVs overlapping these loci and re-ran the enrichment analysis. To identify CNVs for removal, we collated a list of all genes lying in known CNV loci, plus any neighbouring genes whose association signal was also clearly driven by these loci. CNVs hitting one or more of these genes were then removed prior to re-analysis. When analysing deletions all known deletion loci were removed; when analysing duplications all known duplication loci were removed; and when analysing deletions and duplications combined, all CNVs overlapping a known locus were removed irrespective of their class (deletion/duplication).

Calculation of gene set odds ratios

In order to calculate odds ratios for enriched gene sets, the following logistic regression model was fitted to the full set of individuals from each study (i.e. including those in which no large CNVs were identified):

logit (pr(case)) = study + average CNV size + number of CNVs + total number of genes hit + number of genes hit in gene set

where 'average CNV size' is the mean length of all CNVs >100kb for that individual; 'number of CNVs' is the total number of CNVs > 100kb for that individual; 'total number of genes hit' and 'number of genes hit in gene set' count the corresponding number of unique genes hit by these CNVs (any gene hit by two CNVs would only count once). The odds ratio was derived from the coefficient of the 'number of genes hit in gene set' term. Since the unit of analysis is now the individual rather than the CNV, we control for average CNV length and CNV number in line with the recommendations of (Raychaudhuri et al., 2010).

CNV size and number of genes hit as predictors of case-control status

When investigating the relationship between CNV size, number of genes hit and case-control status, the following four models were fitted:

(a) logit (pr(case)) = study + chip

(b) logit (pr(case)) = study + chip + CNV size

(c) logit (pr(case)) = study + chip + total number of genes hit

(d) logit (pr(case)) = study + chip + CNV size + total number of genes hit

Comparing the change in deviance between models (a) and (b), a two-sided test was used to assess the relationship between CNV size and case-control status; likewise, a comparison between (a) and (c) was made for total number of genes hit. Comparison between (c) and (d) was used to assess the relationship between CNV size and case-control status conditional on total number of genes hit, comparison between (b) and (d) giving the analogous result for total number of genes hit conditional on CNV size.

Genes from all CNS annotations with a Bonferroni corrected p-value < 0.05 were combined to create a single associated CNS set (CNS_{SZ}). One such set was created for deletions, another for duplications. A comparison between total number of genes hit and number of CNS_{SZ} genes hit was also performed, with 'number of CNS_{SZ} genes hit' replacing 'CNV size' in the above regression models. Very similar results were obtained when CNS_{SZ} was constructed using only the much smaller 'minimal' subsets of annotations (see above) that capture the bulk of CNS enrichment (data not shown).

Correction for multiple testing

Analyses fall into two main classes, 1) gene set enrichment tests to identify significant associations and 2) subsequent ancillary analyses to investigate the source of any notable enrichment.

1) These comprised primary tests of previously associated gene sets (ARC, NMDAR and FMRP); secondary tests of CNS-related gene sets; and finally tertiary tests of the more comprehensive GO and MGI annotations. At each stage, analyses were performed first for

the combined CNV sample and then for deletions and duplications separately. At each stage of our analysis, gene set enrichment p-values were Bonferroni corrected for the total number of tests performed up to that point, as listed in the table below.

Gene sets	CNV tests	N test (novel)	N test (total)
ARC, NMDAR network, FMRP	Combined, Deletion & Duplication	9	9
CNS-related	Combined, Deletion & Duplication	393	402
MGI (2498 terms) + GO (4026 terms)	Combined, Deletion & Duplication	19572	19974

To test for enrichment with rare, non-synonymous *de novo* mutations from individuals with schizophrenia, the 'minimal set' of terms that capture most of the CNS enrichment signal were collapsed into a single gene set for each of our analyses (combined, deletion and duplication). Results were Bonferroni corrected for these 3 tests. An ancillary analysis was then performed to investigate whether the association signals identified were solely due to ARC and NMDAR genes. As we only explore the source of enrichment signals and do not claim to find novel associations, p-values for these tests are uncorrected. To check that enrichment was not due to some property of NS variants unrelated to disease, the analysis was then repeated using NS *de novo* rare variants identified in unaffected individuals. Results were again corrected for 3 tests. Analyses of the 21 individual gene sets listed in Table S9 were Bonferroni corrected for 3 + 21 = 24 tests.

2) Since ISC and MGS data had previously been used to investigate CNV enrichment for ARC and NMDAR (Kirov et al., 2012), we were interested in investigating whether the enrichment seen in the present combined ISC-MGS-CLOZUK sample was solely due to ISC and MGS. As we are simply investigating the partitioning of the association signal between datasets, it does not make sense to correct for tests performed in the full sample. CLOZUK-only results for the combined CNV analysis and for the analysis of deletions and duplications

separately were corrected for 9 tests.

Prior to discussing CNV enrichment for individual CNS gene sets, we investigate whether the 134 sets as a whole display more evidence of nominal association than would be expected by chance, performing permutation tests at two p-value thresholds separated by an order of magnitude (0.01 and 0.001) (see Table 1). Results are corrected for 6 tests, corresponding to the 2 thresholds x 3 analyses (combined, deletion only and duplication only).

To quantify the effect of removing known loci we employed a permutation test in exactly the same manner, results being given in the lower half of (Table 1). The correction procedure here is identical.

To identify genes contributing most to gene set enrichment we calculated single gene association p-values, listing genes with uncorrected $P < 0.05$ in Tables S4-S6. The number of genes tested in each analysis were: 10200 for the combined analysis, 3918 for deletions and 8759 for duplications, these being the number of genes overlapping at least one contributing CNV. In these tables, single gene enrichment p-values are corrected for the full 10200 + 3918 + 8759 = 22877 single gene tests.

The section investigating correlation between case-control status and CNV size and number of genes disrupted falls outside the two main classes of analysis discussed above. The initial analysis of size and number of genes is corrected for 4 tests (size and number in deletions and duplications), the subsequent 2 tests for CNS_{SZ} are corrected for the full set of 6 tests. Conditional analyses, in which we only explore the source of enrichment signals and do not claim to find novel associations, remain uncorrected.

Supplemental References

Blake, J.A., Bult, C.J., Kadin, J.A., Richardson, J.E., and Eppig, J.T. (2011). The Mouse Genome Database (MGD): premier model organism resource for mammalian genomics and genetics. Nucleic Acids Res 39, D842-848.

Ekholm, B., Ekholm, A., Adolfsson, R., Vares, M., Osby, U., Sedvall, G.C., and Jonsson, E.G. (2005). Evaluation of diagnostic procedures in Swedish patients with schizophrenia and related psychoses. Nordic journal of psychiatry 59, 457-464.

Jakobsen, K.D., Frederiksen, J.N., Hansen, T., Jansson, L.B., Parnas, J., and Werge, T. (2005). Reliability of clinical ICD-10 schizophrenia diagnoses. Nordic journal of psychiatry 59, 209-212.

Purcell, S., Neale, B., Todd-Brown, K., Thomas, L., Ferreira, M.A., Bender, D., Maller, J., Sklar, P., de Bakker, P.I., Daly, M.J., and Sham, P.C. (2007). PLINK: a tool set for whole-genome association and population-based linkage analyses. Am J Hum Genet 81, 559-575.

Wing, J.K., Babor, T., Brugha, T., Burke, J., Cooper, J.E., Giel, R., Jablenski, A., Regier, D., and Sartorius, N. (1990). SCAN. Schedules for Clinical Assessment in Neuropsychiatry. Arch Gen Psychiatry 47, 589-593.
